# Advanced Functional Materials Based on Nanocellulose for Pharmaceutical/Medical Applications

**DOI:** 10.3390/pharmaceutics13081125

**Published:** 2021-07-23

**Authors:** Raluca Nicu, Florin Ciolacu, Diana E. Ciolacu

**Affiliations:** 1 Department of Natural Polymers, Bioactive and Biocompatible Materials, “Petru Poni” Institute of Macromolecular Chemistry, 700487 Iasi, Romania; nicu.raluca@icmpp.ro; 2 Department of Natural and Synthetic Polymers, “Gheorghe Asachi” Technical University of Iasi, 700050 Iasi, Romania

**Keywords:** nanocellulose, nanocrystalline cellulose, nanofibrillated cellulose, bacterial cellulose, hydrogels, nanogels, nanocomposites, drug delivery, wound healing, tissue engineering

## Abstract

Nanocelluloses (NCs), with their remarkable characteristics, have proven to be one of the most promising “green” materials of our times and have received special attention from researchers in nanomaterials. A diversity of new functional materials with a wide range of biomedical applications has been designed based on the most desirable properties of NCs, such as biocompatibility, biodegradability, and their special physicochemical properties. In this context and under the pressure of rapid development of this field, it is imperative to synthesize the successes and the new requirements in a comprehensive review. The first part of this work provides a brief review of the characteristics of the NCs (cellulose nanocrystals—CNC, cellulose nanofibrils—CNF, and bacterial nanocellulose—BNC), as well as of the main functional materials based on NCs (hydrogels, nanogels, and nanocomposites). The second part presents an extensive review of research over the past five years on promising pharmaceutical and medical applications of nanocellulose-based materials, which have been discussed in three important areas: drug-delivery systems, materials for wound-healing applications, as well as tissue engineering. Finally, an in-depth assessment of the in vitro and in vivo cytotoxicity of NCs-based materials, as well as the challenges related to their biodegradability, is performed.

## 1. Introduction

Science and technology continue to move toward the use of renewable raw materials, more environmentally friendly and sustainable resources as a result of their potential to manufacture numerous high-value products with low environmental impact [1,2,3].

Nanocellulosic materials derived from abundant and inexhaustible cellulose are an important component of this vital movement [4,5]. The immense interest generated by these nanomaterials during several decades is mainly related to their exciting properties and possibilities of producing from a multitude of sustainable resources [6].

**Figure 1:**
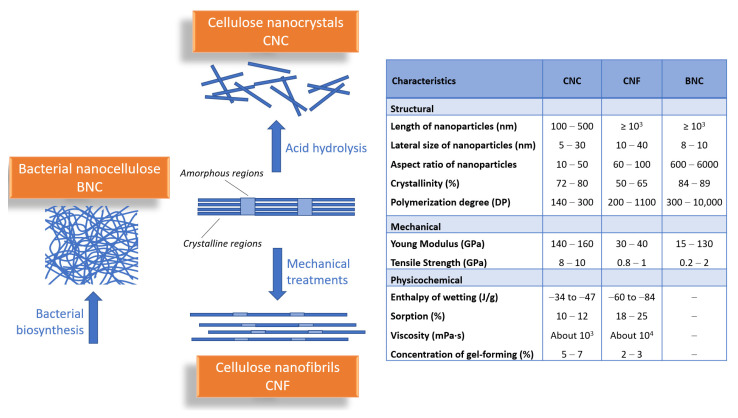
Preparation scheme and dimensions of different types of nanocelluloses: nanocrystalline cellulose, nanofibrilar cellulose, and bacterial nanocellulose.

Generally, cellulose nanomaterials include all cellulose-based materials that are at least one size in nanometer scale, with various shapes, physical properties, or surface chemistry. Depending on the origin of the cellulose, the processing conditions, and the methods of their preparation, nanocelluloses are classified into three main categories (Figure 1): (i) cellulose nanocrystals (CNC), which are short and rigid; (ii) cellulose nanofibers (CNF), long and flexible; and (iii) bacterial nanocellulose (BNC), with high purity and very crystalline [1,7,8,9,10]. Even though these types of nanocelluloses have a relatively similar chemical composition, there are major differences in their degrees of crystallinity and particle size, as well as in their morphological characteristics [11].

Cellulose nanocrystals (CNC), also known as nanocrystalline cellulose (NCC), cellulose nanowhiskers (CNW), or cellulose crystallites, are obtained by acid hydrolysis of wood or various non-wood materials (cotton, hemp, flax, wheat straw, mulberry bark, ramie, Avicel, tunicin, algae or bacteria), which consists in the chemical removal of lignin, hemicellulose and of amorphous regions within cellulose [12]. Cellulose nanofibrils (CNFs), also called microfibrillated cellulose, nanofibrils, and microfibrils, or nanofibrillated cellulose (NFC), are extracted from wood, sugar beet, potato tuber, hemp, flax by delamination of pulp by mechanical pressure before and/or after chemical or enzymatic treatment [9]. *Bacterial nanocellulose* (BNC) or microbial cellulose is produced by some bacterial genera, such as Gram-negative bacteria: *Acetobacter*, *Rhizobium*, *Pseudomonas*, *Salmonella*, etc. or Gram-positive bacteria: *Sarcina ventriculi*, in fermentation processes of sugars and vegetable carbohydrates. This has a large specific surface area, higher water retention value, is considered chemically pure cellulose, and does not contain lignin or hemicellulose [13].

All types of nanocelluloses (NCs) are chemically similar but have different organizational forms and consequently different physical characteristics. As entities, CNFs are micrometer-long fibrils having highly entangled networks of nanofibers with both crystalline and amorphous domains. CNCs are stiffer and highly crystalline rods (ca. 90%), while BNCs are secreted as a ribbon-shaped fibril composed of a bundle of much finer nanofibers.

Beyond possessing the advantageous performances of nanomaterials, the natural nanocellulose (NC) is low cost, completely renewable, highly biocompatible, having low ecological toxicity risk and low cytotoxicity to a range of animal and human cell types compared to the synthetic ones [14,15]. These excellent properties provide NCs an important place in interdisciplinary studies and an increased interest in their applications as biomedical materials.

To date, there has been a strong focus of researchers on the design, manufacture, and processing of nanocellulose-based materials for their potential use in biomedicine, as well as a tremendous increase in the number of scientific publications, from 2015 to 2020, with topic keywords “tissue engineering”, “drug delivery”, and “wound healing”, respectively [16,17].

Thereby, taking into account all this information, the present review provides an overview of the current progress of NCs used as functional materials, with a special focus on their recent pharmaceutical and medical applications, as well as on their toxicological evaluation and in vivo biodegradability.

## 2. Advanced Functional Materials Based on Nanocellulose—General Characteristics

### 2.1. Hydrogels

With the progress of nanotechnology, hydrogels have received much more attention due to their particular and excellent characteristics. Hydrogels were the first biomaterials to be conceived for use in humans. They have moved forward to now mimic basic physiological processes and are essentials as bioactive implants in the sense of “in vivo” scaffolds.

The use of hydrogels as biomaterials is strongly related to their properties. Hydrogels are three-dimensional network colloidal gels from hydrophilic polymer crosslinked able by swelling to absorb and retain large volumes of water in an aqueous environment [18,19,20,21,22,23]. In the swollen state, they have a soft and rubbery structure that mimics the behavior of extracellular matrix (ECM) in biological tissues [24]. Furthermore, hydrogels are conformable to different kinds of surfaces on which they are placed. These properties, in combination with their mucoadhesive nature, elasticity, swelling, and deswelling characteristics in response to environmental stimuli, make hydrogels potential candidates for biomedical applications [18,25]. Thus, hydrogels have found applications to produce different types of materials such as contact lenses [26], blood-contacting hydrogels [27], wound-healing bioadhesives [28], artificial kidney membranes [29], artificial skin [30], vocal cord replacement [31,32], and artificial tendons [33].

Depending on the size of the obtained particles, hydrogels may be classified as macro-, micro-, or nanogels. When they have particle sizes bigger than 100 m, they are usually called macrogels, while gels with particle sizes up to the micrometer range are called microgels. Finally, if these gels are smaller than 100 nm, they are usually considered nanogels [34,35].

### 2.2. Nanogels

Nanogels, also called “hydrogel nanoparticles”, “nanoscalar polymer networks”, “gel nanoparticles”, or “nanoscale hydrogels”, are combine the properties of gels with those of colloids [18]. Generally, they have a spherical shape and size between 20 and 200 nm [36].

Hydrogels, at the nanometer scale, have a great potential in the field of biomedical applications, e.g., as drug-delivery systems, as they combine the characteristics of hydrogels with the advantages of nanoparticles [36,37]. Reducing the size of hydrogel particles in the nano range is reflected in increasing the solubility of hydrophobic drugs, improving the accumulation of drugs in tumors, but also by reducing cytotoxic side effects and increasing the stability of therapeutic agents against enzymatic and chemical degradation [34]. The nanogels also possess some desirable properties, such as high drug-loading capacities, chemical stability, and mechanical properties to avoid the disassembly or fracture during transport, and sensitive response behavior to ensure rapid drug release in response to the relevant stimuli [38].

In addition to their excellent applicability in the drug delivery field, nanogels have also found applications in other biomedical fields, such as chemotherapy [39,40], diagnosis of diseases [41], vaccines delivery [42], biocatalysis [43], and generation of bioactive scaffolds in regenerative medicine [34]. They have also been studied for use in diabetes treatments [44] and gene and protein delivery [41,45].

Nanohydrogels prepared from natural sources have drawn huge attention due to their vast applications in pharmacy, medicine, tissue engineering, cancer therapy, and drug delivery [46]. The use of nanocellulosic materials in obtaining hydrogels from renewable materials has been a much-desired goal that has been achieved for many hydrogel types [47,48,49]. Several smart hydrogels such as injectable hydrogels [33,50,51], shape memory [52,53], supramolecular hydrogels [54,55], double-membrane hydrogels [56], temperature-sensitive hydrogels [57], and many other hydrogels types based on nanocellulose with potential for biomedical applications have been developed.

However, on their own, nanocellulose materials do not gel [58]. Different ways are used to perform, for example, for the gelation of CNC suspensions: by simply increasing the concentration of suspension due to a decrease in the electrostatic double-layer distance [59]; by modifying the solvent conditions through ionic strength increase [60]; by addition of polymers [58]; by sonication [61]; and by hydrothermal treatment at elevated temperature [47].

Nanocrystalline cellulose, with their high rigidity and relatively low anisotropy, are well-suited to act as templates for aligned structures (e.g., artificial muscle-like materials) while providing toughness and flexibility. With collagen, for instance, this afforded networks with mechanical properties similar to tendon and ligaments and excellent biocompatibility [62]. Nanofibrillated cellulose (CNFs) is the type of nanocellulose most likely to form hydrogels due to the length of the nanofibrils. CNFs form gels with much higher elasticity than those resulting from CNCs. CNF suspensions exhibit gelation (G′ > G″, G′ ∝ ω^0^, and G″ ∝ ω^0^, where, G′ is the storage modulus, G″ is the loss modulus, ω is the frequency) even down to a concentration near to 0.1 wt.%, i.e., the critical gelation concentration, above which the nanofibrils form interconnected networks [63]. CNFs will afford such structures at concentration ranges of 0.05–6 wt.% [62]. The simplest case is offered by pristine CNFs, which spontaneously form hydrogels, probably promoted by their length and interacting entanglements [64].

### 2.3. Nanocomposites

Currently, research is also progressing in the field of nanocomposite hydrogels, including functionalized nanomaterials [18]. In general, in order to improve or modify certain properties, polymeric matrices of nanocomposites are reinforced with nanoparticles/nanofillers [65]. In particular, hydrogels are reinforced with nanoscale materials to obtain nanocomposites with high mechanical strength characteristics or are combined with nanoparticles that confer antibacterial or magnetic properties [34].

#### 2.3.1. Nanocellulose Materials as “Reinforcing Agents” into Polymer Matrices

Over the past decade, composite materials have attracted a great deal of interest, and particular attention has been focused on the use of nanocellulose as an alternative to inorganic reinforcing agents in polymer matrices for the production of fully “green” composites [66,67,68]. Nanocellulose, owing to its exceptionally high mechanical properties (high specific strength and modulus), high surface area, high aspect ratio, and low environmental impact, has greater advantages as reinforcing filler in comparison to glass fibers, silica, carbon black, and other expensive nanosized fillers [65]. Thus, composite materials with natural fillers have not only met the environmental appeal but also contributed to developing low-density materials with improved properties [69].

Hydrogels entirely made of biopolymers and reinforced with nanocellulose can be classified as “green” nanocomposite materials because of their renewable and biodegradable design [70]. The design of cellulose-based biocomposites is a pathway with many alternatives due to the wide variety of cellulose fibers with specific geometries, the diversity of polymers and manufacturing processes, the multitude of types of reinforcements, and the possibilities of orientation and arrangement of fibers [17].

Nanocrystalline cellulose has been investigated as reinforcing agents for a variety of polymeric systems due to their large aspect ratio, high specific strength and modulus, low density, high surface area, and unique optical properties [51,62,65,71,72]. CNCs have been used as reinforcing agents in a wide range of polymer matrices, from the most common to the most unusual, such as: poly(vinyl alcohol), poly(oxyethylene), polyethylene glycol, poly(*N*-isopropylacrylamide), starch, natural rubber, or polyurethane [67,73,74,75,76].

Nanofibrillated cellulose (NFC) has excellent properties for mechanical reinforcement due to its special morphology that combines the advantages of the length of the fibers in the micrometers range with those of their width in the nanometers range [69]. The use of CNF networks as reinforcing elements together with a suitable matrix polymer is an efficient reinforcement solution for high-quality, specialized applications of bio-based composites. The combination of nanofiber flexibility, aspect ratio, and strength is the main advantage of CNFs in various applications [77]. For instance, comparing the reinforcement capacity of CNC and NFC (at the same addition) using poly(ethylene oxide) (PEO) as the polymer matrix, Xu and coworkers [75] reported that the nanocomposites reinforced with NFC demonstrated higher-strength and elastic modulus than nanocomposites with CNC. However, CNC-based nanocomposites presented a higher strain of failure. The higher strength values of NFC-based nanocomposites are the effect of their high aspect ratio of cellulose nanofibrils that favors more entanglement and network percolation.

By comparison, bacterial nanocellulose (BNC), having the highest purity of all nanocellulose materials, doubled by high crystallinity and excellent biological affinity, is the ideal reinforcing component for biopolymer composites [78].

#### 2.3.2. Nanocellulose Materials as “Matrices” for Different Reinforcing Agents

The inclusion of biocompatible and/or bioactive compounds as components of the composite is the proper way to overcome certain limitations of nanocellulose materials, improving their biocompatibility, antimicrobial activity, or water-holding capacity [79].

Generally, nanocellulose can be reinforced with different polymers with specific properties, obtaining a material with different characteristics from those of starting materials [65]. Nanocellulose can also be used as a substrate for the incorporation of inorganic nanoparticles, such as carbon nanotubes, graphene, and graphene oxide to obtain hydrogels with antibacterial, antiviral, antifungal, magnetic, electrical, and mechanical properties. The high specific surface area, the presence of reducing functional groups, and the ability to form aqueous suspensions are the main arguments for the use of nanocelluloses as a support for metal/metal oxide nanoparticles [80]. The process of composite formation is performed through physicochemical interactions or by mechanical capture of nanoparticles in the structural matrix of nanocellulose [81].

Nanocellulose-based compounds have found applications in various biomedical areas, from dressings to drug administration and even as a basis for scaffolding in regenerative medicine [82]. Silver particles have been used as potential agents with a broad antibacterial activity and low presumed toxicity to coat cellulosic materials for biomedical applications. The composite was prepared by immersing BNC in a silver ammonium solution and showed to be effective as dressing in wound-healing applications by decreasing inflammation and promoting wound-healing [30]. The silver nanoparticles were incorporated in crystalline nanocellulose by microwave-assisted synthesis, and the composites proved to have high antibacterial properties against *E. coli* (Gram-negative bacteria) and *S. aureus* (Gram-positive bacteria) [83]. Barua and coworkers [84] prepare copper-copper oxide nanoparticles (Cu–CuO) NP-coated CNFs through a green reductive technique, which exhibited promising antimicrobial activity against Gram-positive and Gram-negative bacteria and fungal species.

## 3. Nanocellulose-Based Materials in Pharmaceutical/Medical Applications

The nanocellulose materials, used as independent functional material or as reinforcement units in composite materials, have received tremendous attention in a wide variety of applications, including foods, packaging, cosmetics, biomedical implants, optics, water filtration, hygienic applications, and so forth [3,14,17]. However, especially in biomedical fields, they appear to have significant advantages due to their intrinsic biodegradability and biocompatibility [14]. However, other interesting features should also be considered, such as mechanical properties, low risk of cytotoxicity, its three-dimensional (3D) nanofibrous network, and last but not least, its natural source [62,85,86].

### 3.1. Nanocellulose-Based Materials in Drug-Delivery Systems (DDS)

An ideal drug carrier should be nontoxic, non-immunogenic, biocompatible, and biodegradable; enhance drug solubility and stability and have high drug-loading capacity; and be capable of reaching correct concentrations at a proper rate determined by an optimal [36]. Other equally important criteria to be met are related to its size and surface characteristics because these two parameters control the residence time in the bloodstream and the target site. More exactly, the size needs to be sufficiently large enough to prevent rapid penetration into fenestrated blood vessels, yet sufficiently small to avoid phagocytosis. The surface nature also decides the duration and destination of the drug carrier in the circulatory system. For instance, a hydrophilic surface will most likely make the carrier avoid phagocytosis by macrophages, and this hydrophilicity can be accomplished either by covering the surface with a hydrophilic polymer (i.e., PEG) or by using block copolymers with hydrophilic and hydrophobic areas [62].

Being a natural nanosized material, nanocellulose features meet the necessary criteria mentioned above regarding its function as a vehicle for DDS: its horizontal measurements extend from 5 to 20 nm, and the longitudinal measurement ranges from 10 nm to a few microns; each of its monomers bears three hydroxyl groups with the ability to form hydrogen bonds, which plays a major role in the surface hydrophilicity. Of course, the nanocellulose unique properties should not be overlooked because they make this material play an important role among drug-delivery vectors, such as high crystallinity, biocompatibility, biodegradability, high surface area, unique mechanical and rheological properties, liquid absorption capacity, and porosity [36,85,87,88].

The three types of nanocellulose are quite similar to each other, yet there are distinct differences that set them apart. These differences make each type of nanocellulose better suited for a certain drug-delivery system compared to the others [87]. We summarize the recent reports on nanocellulose-based drug-delivery systems in Table 1.

#### 3.1.1. CNC-Based Materials in Drug-Delivery Systems

Crystalline nanocellulose (CNC) has gained interest as a promising nanomaterial, with potential applications both as drug-delivery systems and in diagnostic medicine [91]. When cellulose undergoing acid hydrolysis using sulfuric acid, around a tenth of the carboxyl group at the surface is sulfated, resulting in CNCs with a net negative charge, and this represents a considerable advantage of CNCs in the delivery of positively charged therapeutic compounds [62]. However, it was found that CNC alone has a reduced ability to function as a drug carrier, so different surface modification techniques, including physical absorption and chemical grafting, are necessary to improve this behavior [24].

Chitosan oligosaccharide (CS_OS_) (Mn 5500 Da) was grafted on CNCs surface-functionalized with carboxyl groups by TEMPO reaction (CNC-OX) [90]. Hybrid hydrogel (CNC-CSOS) was tested as a controlled release system for two drugs, namely procaine hydrochloride (PrHy) and imipramine hydrochloride (IMI). In vitro drug-release studies were conducted in PBS buffer and demonstrated a slower release compared to non-grafted CNC. For instance, the total amount of PrHy released was the lowest for CNC-CS_OS_, namely 40%, but in a more sustained manner, proved by the maximum release time around 12 min, compared with pristine CNC (~80% in about 6 min) and CNC-OX (~60%, ~3 min). This could be explained by the presence of chitosan chains on the surface of CNC that inhibited the release and diffusion of the drug molecules. Moreover, different release profiles at different pH values were obtained. The sustained-release profile for PrHy was observed at pH 6 and for IMI at 7.4, respectively. In addition, a burst release for IMI appeared at a pH of 5.7 [90].

Folic acid (FA) was grafted on CNCs for the first time by Dong and coworkers [103], and the synthesized conjugate was used for the targeted delivery of chemotherapeutic agents to cancer cells. The folate-receptor-mediated uptake was explored in vitro by human and rat brain tumor cells. In cell uptake studies, CNCs were labeled with fluorescein isothiocyanate (FITC) followed by conjugation with FA. In vitro results indicated that the cellular uptake of FITC-CNC-FA by the folate receptor was considerably superior to free FA. This study suggests that the FA-conjugated CNCs selectively target the folate-receptor-positive cancer cells and are promising candidates for potential cancer targeting.

Hydroquinone-cellulose nanocrystal complex, prepared by incubating hydroquinone solution in CNC suspension, was developed for topical delivery of hydroquinone (HDQ) to inhibit the production of melanin and prevent discoloration of the skin [91]. Although HDQ is susceptible to oxidation and rapidly darkens in the presence of oxygen, in the CNC-HDQ complex, there was no sign of darkening after 1 week, proving the CNCs could protect HDQ from oxidation. In the first hour, 40% of the HDQ was released from the HDQ-CNC complex, and in the next 4 h, it was sustained released and much slower an amount of about 80% of the bound HDQ, in the aqueous medium. This sustained-release behavior from the HDQ-CNC complex demonstrated the potential applicability of CNC as a suitable drug carrier for drug administration to the skin.

**Figure 2:**
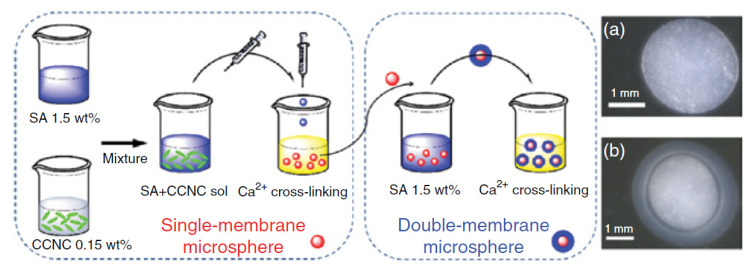
The preparation routine of single-membrane and double-membrane microsphere hydrogels. Optical microscope images of (**a**) the SA/CCNC single-membrane microsphere hydrogel and (**b**) the SA/CCNC-1 h double-membrane microsphere hydrogel. Reproduced with permission from [89]. American Chemical Society, 2016.

Double-membrane hydrogels were obtained from cationic cellulose nanocrystals (CCNC) and anionic alginates, and the preparation technique of single-membrane and double-membrane microsphere hydrogels is shown in Figure 2. Two kinds of drugs (an antibiotic and a grow factor) were introduced into the different layers of the double-membrane hydrogels: the ceftazidime hydrate (CH) was added in the external membrane of pure alginate and the epidermal growth factor human (EGF) in the internal membrane of CCNC-alginate, respectively [89].

The different compositions and properties of the internal and external membranes led to a separated and ordered drugs release from the hydrogels: the CH molecules were rapidly released within the first three days at pH 7.4, while no signal of the EGF release can be detected. The rapid CH release can be attributed to the swelling/disintegration mechanism that takes place on the external alginate-based layer of the hydrogel: at the beginning, the outer membrane underwent swelling and absorbed water during the first day (proved by the thickness and transparency increase in the external membrane); then, a gradual reduction in the swelling ratio from the first to third day occurs, attributed to the erosion process that now controls the drug release. On the other hand, the EGF release started after four days and showed a different release behavior, with a prolonged release lasting for 7–8 days and reaching equilibrium at the eleventh or twelfth day. It seems that cationic cellulose nanocrystals, due to their nanostructural construction, can provide a “nano-obstruction effect” to prolong the drug release for the partial disintegration, and meanwhile play a “nano-locking effect” to further prevent the burst release of the drugs during the gradual disintegration of the hydrogel. These hydrogels with special double-membrane structure and alternative drug-release behaviors have the potential to be used in oral administration and wound-dressing applications [89].

Different nanocomposites with a promising role in drug-delivery applications were obtained by using CNCs as a reinforcing agent for different biopolymers, such as chitosan, gelatin, and alginate or quaternized cellulose. pH-sensitive chitosan hydrogels reinforced with CNCs (CS/CNC) were synthesized using chemical crosslinking and tested for drug release and delivery of curcumin in SGF (simulated gastric fluid). An addition of 0.5% CNCs determine a 50% increase in the compression strength of CS hydrogel (from 25.9 ± 1 to 38.4 ± 1 kPa), and the maximum swelling ratio was found at pH 4.01. The drug-release profile of hydrogels was directly influenced by hydrogels swelling degree, the highest release being indicated by 0.5% CS/CNC hydrogel (0.74 ± 0.03 mg/L). In addition, as expected, it decreases with increasing reinforcing agent content. The curcumin release kinetics was investigated with the Ritger–Peppas model [104]. The values obtained for the release of curcumin in SGF, corresponding to the exponent, are *n* = 0.61 and 0.76, which indicates that the diffusion and release of the drug take place in a non-Fickian (anomalous) manner when both diffusion and erosion processes control the release of the drug. Due to its pH sensitiveness, the chitosan hydrogels reinforced with CNCs can be a promising candidate for stomach-specific drug delivery [92].

**Figure 3:**
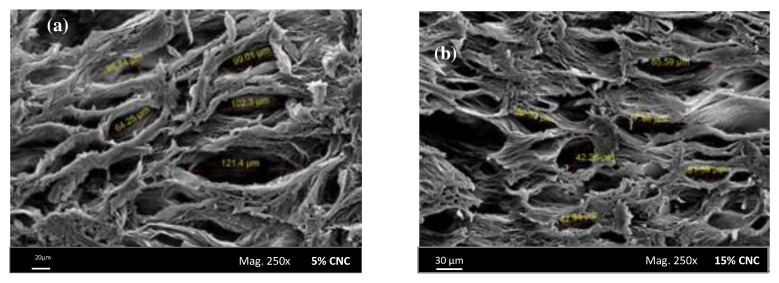
SEM images of the hydrogels with 5% CNC (**a**) and 15% CNC (**b**). Reproduced with permission from [94]. Elsevier, 2015.

pH-sensitive gelatin hydrogels reinforced with CNCs were prepared by casting techniques using glutaraldehyde as a crosslinker. CNC nanoparticles were incorporated into gelatin hydrogels in different ratios, between 5% and 25%, in order to improve their mechanical and thermal stability. Figure 3 shows the SEM images of the hydrogels with 5% and 15% of CNCs, respectively, and it was found that the voids in the gelatin network were gradually filled with the increase in CNC into the gelatin network. In order to evaluate the potential of these hydrogels as drug carriers, theophylline was used as a model drug. The drug-release pattern for all hydrogels tested can be divided into two phases: the first phase is the burst effect corresponding to the early release of drug molecules present on the surface of the hydrogel, and the second phase, which is a continuous drug-release process. Although the 5% CNC-gelatin hydrogel showed the highest drug-loading efficiency (Table 2), the drug-release rate was too high, approximately 70% of the drug releasing in the first 4 h. At the other extreme, the rate of drug release was too slow for the 20% and 25% CNC-gelatin hydrogels, with a large quantity of unreleased drugs remaining after 24 h. The gelatin hydrogels reinforced with 15% CNC were considered the best potential candidates for a controlled drug-delivery system, showing a suitable balance between drug-loading efficiency and controlled drug-release behavior [94].

Using the in situ gelling method, nanocomposite hydrogels were made by crosslinking in the presence of β-glycerophosphate (β-GP) of quaternized cellulose (QC) and rigid rod-like cationic cellulose (CCNC) nanocrystals. The incorporation of CCNCs (1; 1.5, and 2.5 wt%) into the QC/β-GP system was an effective way to improve the mechanical properties of the hydrogels (2.5 wt.% CCNCs doubles the value of hydrogels storage moduli). Doxorubicin (DOX) is a model anti-cancer compound, often used in various studies, and in this case, the nanocomposite hydrogels incorporated with DOX were injected in the vicinity of mouse tumors containing liver cancer xenografts. In all cases, excepting the pure QC/β-GP system, the burst effect was not observed during the initial stage of DOX release. With the introduction of CCNCs, the DOX release from hydrogels nanocomposites with 1% to 2.5% CNCs continued in a more sustained-release manner compared to that of hydrogel without CNCs. For instance, the hydrogel with 2.5 wt.% CCNC loading could sustain release for at least 17 days the delivery rate of DOX was about 29.4 μg/day. Moreover, considering the nature of the release mechanism being surface erosion-based, one can approximate the constant release profile of these hydrogels in the zero-order release kinetics. Compared with the control group (saline), when all the mice died within 12 days after the operation, the administration of DOX-loaded hydrogels suppressed the tumor progression significantly and increased the survival rate of the mice. The nanocomposite hydrogels based on quaternized cellulose and cellulose nanocrystals proved to have great potential for application in sustained delivery of anti-cancer drugs in order to increase therapeutic efficacy [93].

#### 3.1.2. BNC-Based Materials in Drug-Delivery Systems

Bacterial nanocellulose-acrylamide (BNC-Am) hydrogels were synthesized using microwave irradiation technique on mixtures of Am and BNC in NaOH-urea solution, containing *N*,*N*-methylene-bis-acrylamide (MBA) as crosslinker. The mixture Am/BNC was only dispersed or total solubilized in NaOH-urea solution. The hydrogels exhibited a low swelling ratio in acidic medium (pH 2–5), which increases with increasing pH until reaching a peak at pH 7, proving the pH-responsiveness property of the synthesized hydrogels. Furthermore, these hydrogels were tested in controlled delivery of theophylline, as a model drug, at pH 7.4. The release profiles of all hydrogels into PBS solution (pH 1.5 for 2 h followed by pH 7.4 for up to 48 h) were best fitted by zero-order release kinetics, with the release rates of solubilized BC-Am hydrogels higher than dispersed BC-Am ones. The acute oral toxicity tests on 8-week-old female mice, Institute of Cancer Research (ICR) mice, showed that hydrogels are nontoxic, even at an oral administration of 2000 mg/kg and no histopathological changes were recorded compared to control mice. The results proved that developed pH-sensitive hydrogels have the potential to be used in oral drug delivery [25].

A dual stimuli-responsive system (pH, electric) consisting of hybrid hydrogels of BNC-sodium alginate (SA) was investigated for swelling and controlled release of ibuprofen as a model drug. The hybrid hydrogels were obtained by homogeneous dispersion of BNC in SA and crosslinked with CaCl_2_. It was recorded an increase in the swelling ratio as a function of pH, so at a low pH (1.5), the hydrogel swelled less than 8 times compared to its dry weight, while at an increase in pH (11.8), the swelling of hydrogels increased up to 13 times more. By protonation and deprotonation of calcium alginate, the release of ibuprofen was pH-controlled (faster in alkaline, slower in acidic). The drug released amount reached more than 90% in the alkaline medium and approximately 60% in the acidic medium at the end of the release process (30 h). Thus, the BNC-SA hybrid hydrogels exhibited a characteristic pH-sensitive release behavior, a faster release in neutral and alkaline medium and slower in acidic medium, respectively. Moreover, the release of drugs in pH 11.8–1.5 was fitted with the Korsmeyer–Peppas model [105]. The exponent “*n*” revealed that the drug-release mechanism in pH = 11.8 was more diffusion-based (*n* = 0.498) than the one in pH = 7.0 (*n* = 0.702) or pH = 1.5 (*n* = 0.77), respectively. Therefore, the results indicated a drug release controlled by a non-Fickian diffusion mechanism. Under an applied electric field (0–0.5 V), the swelling ratio of the hydrogels increased from 8 to 14 times the dry weight, and the drug release was enhanced. For instance, after 8 h, the released drug amount at 0.5 V was twice higher than that of 0 V, and it took approximately 12 h to obtain a drug release of about 90% at 0.5 V. Furthermore, the drug-release mechanism under applied electric voltages was controlled mainly by the diffusion process (*n* = 0.4919), and the Korsmeyer–Peppas model can be applied to explain the release profiles of BC/SA hybrid hydrogels [99].

The release behavior of ibuprofen sodium salt (IbuNa) used as a model drug was analyzed also using another type of composite hydrogels based on BNC and carboxymethylcellulose (CMC) using epichlorohydrin as a crosslinking agent with two different concentrations, 2.5% and 7.5%, respectively. The BNC membranes were immersed in CMC solutions with different concentrations 0.1, 0.15, 0.2 and 0.25% for 24 h. In vitro drug-release studies from BC-CMC hydrogels were performed investigating the effect of CMC composition and crosslinking. An aqueous buffer of pH 7.4 was chosen as a drug-release medium because it simulates the condition of intestinal fluid. The swelling and drug-releasing properties of hydrogels are equally influenced by the concentration of CMC and epichlorohydrin. Therefore, an increase in CMC and crosslinking concentration leads to an increase in gel fraction. The maximum gel fraction value (GF = 92.499% ± 0.010%) was obtained with the highest CMC content and crosslinker amount. The increase in epichlorohydrin concentration conducted to hydrogels with a higher degree of crosslinking and finally leads to lower values of swelling ratio and lower values of cumulative release. The drug-release pattern for all hydrogels tested includes a burst phase in the first 2 h, with a drug release between 55% and 83% depending on the crosslinking degree of hydrogels, followed by a more sustained release lasting up to 24 h. Although they are preliminary, these results suggest that BC-CMC composite hydrogels could be used in drug delivery [106].

### 3.2. Nanocellulose-Based Materials in Wound-Healing Applications

Human skin is the largest organ in the human body, with the primary function of acting as a vital barrier to provide protection against adverse effects of the surrounding environment (microorganisms, mechanical impacts, hazardous substances, and radiation). In addition, it is an organ of protection, sensation, and regulation (e.g., body temperature, moisture) [27,107]. A break in the skin affects all the functions of the organ, and therefore, any wound must be rapidly and efficiently repaired. Wound healing represents a series of biological processes in monocytes, and macrophages play a vital role in order to restore skin functions [108] to prevent dehydration and minimize the risk of bacterial infections: shortly after injury, monocytes reach the wound site, differentiate into macrophages, and participate in the phagocytosis of necrotic tissue and secretion of cytokines and growth factors; macrophages release a wide spectrum of soluble mediators that recruit and activate fibroblasts and promote angiogenesis and epithelialization [27]. Most of the skin lesions that result in minimal tissue damage heal efficiently within a week or two. However, severe injuries require outside intervention to minimize the risk of infection or dehydration of the wound bed [27], such as different types of wound dressings based on natural or synthetic polymers, as well as their combinations. The wound dressing could have different forms (films, foams, membranes, and hydrogels), and most of the time, they contain bioactive substances such as drugs, growth factors, peptides, and others, which can accelerate wound healing [79].

**Figure 4:**
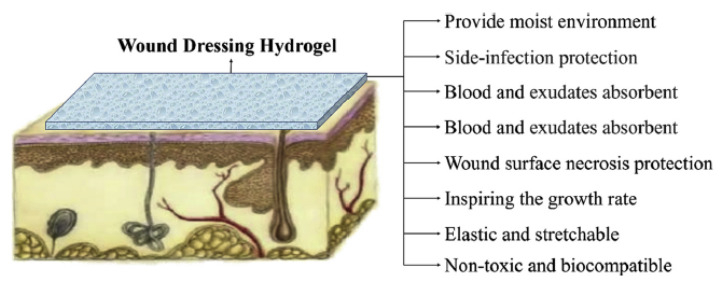
Schematic illustration representing the application of hydrogel materials in the wound-dressing field. Reproduced with permission from [24]. Elsevier, 2019.

The requirements of an “ideal wound dressing” are quite challenging: must provide a moist environment, oxygen circulation, ensure liquid drainage, absorb the excess of fluids that exudes from the wound, and provide wound protection from infections. In addition, it must be easy to apply and painless to remove. It should be biocompatible, have enough mechanical strength to avoid undesirable fracture, could accelerate the healing process, and restore the structure and function of the skin (Figure 4) [38,79,109,110]. Among different wound-dressing materials, hydrogels have received special attention owing to their unique characteristics such as super-high absorbability, ability to provide hydration for healing, suitable oxygen permeability, easy replacement, and controlled drug release [24,111].

Hydrogels match most of the challenging features mentioned above, being the most appropriate material for wounded patients. In addition, the hydrogel can encapsulate actives substances (i.e., antibiotics) in their molecular network during gelling and release them in a controlled manner at the wounded site [18]. However, the mechanical properties are essential in wound dressing, and therefore, the inherent mechanical weakness of many hydrogels remains the major drawback in such applications. Several approaches have been employed in order to improve the mechanical properties of hydrogels, and one of them would be the use of hydrogels with nanosized particles (i.e., cellulose nanoparticles) or incorporation of cellulosic polymers to the hydrogel’s network for preparing versatile and robust wound dressings [38,112].

Nanocellulosic materials have superior mechanical properties and a high water absorption capacity, which allows the skin to maintain its natural moisture and to absorb the exudate from wounds, where appropriate, properties that show the resemblance to the nanoscale architecture of the extracellular matrix (ECM) [107]. Last but not least, nanocellulose contains numerous hydroxyl groups, which facilitate the mucoadhesion with epithelial tissues and also facilitate the formation of composites hydrogel with other polymers or small molecules, which can inhibit the propagation of bacteria on the surface of a wound [38].

#### 3.2.1. CNC-Based Materials in Wound-Dressing Applications

Crystalline nanocellulose (CNC) application in wound dressing mainly refers to its nanoparticles used as a reinforcing material for a series of natural or synthetic polymers, such as chitosan, gelatin, poly(*N*-isopropylacrylamide), or polyvinyl alcohol (PVA), which possess certain properties suitable for wound dressing but lack the mechanical characteristic.

Zubik et al. [112] evaluated the application of CNCs as a reinforcing material in obtaining injectable hybrid hydrogels based on poly(*N*-isopropylacrylamide) (PNIPAAm). The hybrid hydrogels were prepared by free-radical polymerization without using any additional cross-linkers. They combine the stimuli-responsiveness of PNIPAAm with the incomparable mechanical properties of CNCs nanoparticles. Thus, the PNIPAAm-CNC hybrid hydrogel with the highest CNC content (50 mg/mL water) exhibited the best mechanical property, the optimum VPTT (volume phase transition temperature) value in the range of 36 to 39 °C, which is close to normal human body temperature, have the highest ESR (equilibrium swelling ratio) value, and the greatest stability after injection. Furthermore, for wound-dressing purposes, this mechanical stable and hydrogel with thermo-responsive behavior were selected for drug-loading study, more exactly for in vitro release of metronidazole, an antibiotic used in wound infections. The selected PNIPAAm-CNC hybrid hydrogel proved its suitable drug-loading capacity at room temperature and a burst drug release followed by a slow and sustained release at 37 °C. Therefore, these PNIPAAm-CNC hybrid hydrogels are promising materials to be used as injectable hydrogel for wound dressing [112].

Polymeric blends of chitosan (C) and gelatin (G) with different amounts of nanocrystalline cellulose (NCC) and calcium peroxide (CP) particles were prepared by [113]. Chitosan and gelatin films have potentials in wound-healing applications but have unstable structures in an aqueous state, which is improved by adding NCC nanoparticles. The mechanical resistance of polymer films increases with increasing NCC concentration, both for 0.3 and 1 wt.%, and the polymer blends remain stiff enough even at wet states. In order to accelerate wound healing, CP particles were added into the polymer mixture, which was used as oxygen-releasing material. CP particles are used against E. coli bacteria when it has been established to have antibacterial activity. The process of oxygen release reaches its maximum value on the first day and has a constant value for 7 days. Further, an MTT assay was used to evaluate the influence of CP or NCC on the fibroblast cell proliferation onto polymer films. The lack of cytotoxicity of polymeric films was demonstrated by increasing the number of viable cells after 3 days, for all samples, with a significant increase recorded after 7 days. In addition, it was observed that the cell viability of NCC-containing films was higher than that of C-G control, explained by the similarity of NCC structure with ECM, which leads to adhesion of fibroblast cells on the surface of the film. Secondly, the growth of cells was significantly increased by the addition of CP to the film composition, and this can be attributed to the oxygen release ability of CP after placement in a cell culture medium, which appears to improve cell viability. In conclusion, it would be expected that, using NCC together with CP as a source of oxygen, cell growth has remarkably increased [113].

Cellulose nanowhiskers (CNW) have been incorporated into polyvinyl alcohol (PVA) hydrogels in order to obtain nanocomposite hydrogels with improved properties and the possibility of being used in wound-dressing applications. In this sense, it was proved that the incorporation of CNW in the PVA hydrogel matrix causes significant improvements in mechanical properties, an increase in thermal stability, and allows the control of pore morphology, thus maintaining the transparency of the samples. In addition, there was a rate of water vapor transmission that includes hydrogels in the field of application of dressings and a microbial penetration that recommends them as suitable barriers against various microorganisms. The results showed that even a 3 mm thick nanocomposite hydrogel dressing could protect the wound from bacterial penetration, compared to 64 layers of gauze, which cannot prevent exogenous bacteria from entering the wound. All these results confirm that PVA/CNW materials have the potential to be used as a wound dressing [68].

#### 3.2.2. NFC-Based Materials in Wound-Dressing Applications

Nanofibrillated cellulose (NFC) applications in the wound-dressing field are presented in more detail below. NFC hydrogels crosslinked in the presence of calcium ions were investigated as potential materials for wound healing. The choice of calcium ions was based on their properties as a hemostasis cofactor and modulator in epidermal cell regeneration [114]. The water retention tests showed that hydrogels have the potential to maintain a suitable moist environment at the wounds. The biocompatibility of the crosslinked NFCs was investigated by indirect cytotoxicity tests using human dermal fibroblasts (hDF) and monocyte-like THP-1 cell lines, and also by direct cytocompatibility test with hDF cells, studying the inflammatory response of blood-derived human mononuclear cells. Cell viability was above the 70% toxicity limit, more exactly 78% ± 3% for hDF cells and 104% ± 6% for THP-1 cells, respectively, which indicates no toxic effects for hydrogels due to the leaching. The nature of hydrogels has been studied as an inflammatory response with blood-derived mononuclear cells in relation to the secretion of cytokines when the hydrogel has been shown to be inert [27].

Xu Chunlin and collaborators [115] presented, for the first time, an approach to 3D printing of TEMPO-oxidized CNF hydrogel scaffolds with one component only. The printed scaffolds were subjected to double crosslinking: first, during printing by Ca^2+^ cations, and second, post-printing by chemical crosslinking with 1,4-butanediol diglycidyl ether (BDDE). The ratios (*w*/*w*) between BDDE and CNF corresponded to a low crosslinking degree (0.01) and high crosslinking degrees (0.18), respectively. Biocompatibility of the scaffolds as well as adhesion and proliferation of human dermal fibroblasts (HDF) cells isolated from human foreskin were assessed. The cell tests confirmed that printed scaffolds are nontoxic and, compared with control 2D structures, 3D printing showed great HDF cell viability. The cell adhesion on the 3D printing matrix, measured after 12 h of incubation, was slightly lower compared with 2D control samples, and the matrix thickness has a great influence: the 3 mm samples have a higher percentage of cell attachment (84%–86.5%) than 2 mm ones (78.25%–79.25%). In conclusion, the suitable biocompatibility and only a slight inhibition of cell adhesion provide suitable opportunities for the safe application of these materials in wound-healing therapies [115].

The potential use as a wound-dressing material, of a dressing-based on NFC from wood, was examined in a clinical study on a group of nine burned patients [108], and its efficiency was compared with commercial lactocapromer dressing, Suprathel^®^. After 28 days, this study allowed drawing some conclusions regarding the NFC material as wound dressing, namely: NFC dressing adhered well to the wound sites and self-detached from the wound surface after 11–21 days for all nine patients, which is faster compared with Suprathel^®^ that self-detached after 16–28 days for patients 5–9; no allergic or inflammatory response to NFC was observed. Regarding the antimicrobial properties of NFC wound dressing, it was observed that NFC does not have antibacterial properties against *Staphylococcus aureus* and *Pseudomonas aeruginosa* (the common wound pathogens), but neither do they support its growth. In conclusion, NFC seems to be a promising material to obtain skin grafts since it is biocompatible, attaches easily to the wound site, and remains in place until the wound is healed [108].

NFCs-gelatin (G) structure was developed by Raghavendra and coworkers [116], for controlled release of nanocurcumin (NC), in order to be used for wound dressing and antimicrobial applications. Nanoscale curcumin material is developed based on an aqueous solution by ultrasonication process. The size of nanocurcumin was in the range of 2–15 nm, proved by TEM analysis. The releasing studies showed a slow and sustained-release pattern for about 60 h for NC-G-NFCs, suggesting the effectiveness of the material in wound care for a longer period of time. Regarding antimicrobial properties, a comparative study was performed for nano-curcumin impregnated gelatin-cellulose fibers (NC-G-NFCs) and curcumin impregnated gelatin-cellulose fibers (C-G-NFCs) against *E. coli* and *S. aureus*. Although inhibition zones of 2.5 to 6 mm were observed in all samples, the superiority of the antimicrobial performance of the NC-G-NFC sample was evident. This may be due to the presence of quantitatively and stoichiometrically more nanocurcumin molecules in NC-G-NFCs structure than C-G-NFCs, which are involved in the bacterial devastation process. Hence, NC-G-NFCs prepared completely from naturally available materials can be considered as a novel kind of functional material for wound dressing and antimicrobial applications [116].

**Figure 5:**
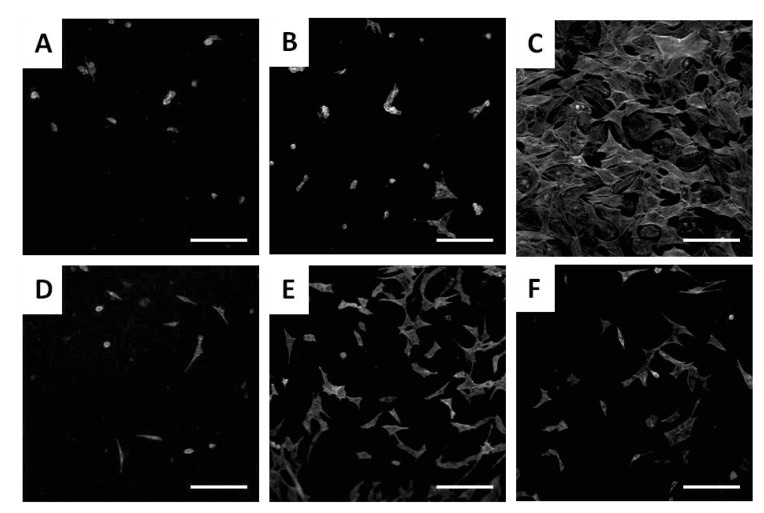
Confocal laser scanning images of C3H10T1/2 cells on Ca^2+^- and Fe^3+^-crosslinked NFC hydrogels: (**A**) unmodified Ca^2+^-NFC hydrogel; (**B**) Ca^2+^-NFC hydrogel with physically adsorbed PF; (**C**) Ca^2+^-NFC hydrogel covalently attached PF; (**D**) unmodified Fe^3+^-NFC hydrogel; (**E**) Fe^3+^-NFC hydrogel with physically adsorbed PF; (**F**) Fe^3+^-NFC hydrogel with covalently attached PF. (Scale bar denotes 200 μm). Reproduced with permission from [117]. American Chemical Society, 2014.

Zander and coworkers studied the viability and proliferations of C3H10T1/2 fibroblast cells onto Ca^2+^ and Fe^3+^-crosslinked carboxylated CNF hydrogels [117]. The protein fibronectin (PF) adsorption, physically and chemically, onto the hydrogel surface was necessary to improve cell adhesion because the nanocellulose lacks biorecognition, although it supports cellular growth. Morphological studies on both control and modified hydrogels highlight a network structure made of interconnected nanofibers, which have open pores. C3H10T1/2 cells were grown on the surface for 5 days, and the best cellular attachment and spreading was observed on the Ca^2+^ crosslinked gels with covalently attached PF, followed by the Fe^3+^ crosslinked gels with physically adsorbed PF (Figure 5). The cellular adhesion was minimal on unmodified hydrogels, and in all cases, improved with the PF attachment. The percentage of cells spread was higher for Fe^3+^ crosslinked gels compared to the Ca^2+^ gels (except for the Ca^2+^ gel with covalently attached PF), possibly due to the increased hydrophobicity and greater adsorption of serum proteins. However, this behavior can also be attributed to the certain toxicity of calcium ions, proven in a recent study by Meschini et al. [118], where Ca^2+^ was used as a gelling agent of negatively charged cellulose nanocrystals along with natrium and magnesium ions. However, as mentioned in the study, this high toxicity can be avoided if the excess of calcium cations from hydrogels are removed by appropriate rinsing or soaking treatments, and more attention is paid to the manufacturing process. In their current state, the hydrogels still possess many of the desired characteristics for a tissue substrate and, with protein modification, successfully supported the growth of fibroblasts. Thus, these hydrogels are suitable for biomedical applications in which cellular infiltration is less critical such as bandages or wound-healing materials. In addition, due to their high surface area and porosity, they could also find applications in drug delivery, filtration, and catalysis [117].

Brown algae nanofibrilated cellulose (BA-CNFs) was used to prepare sponges with high porosity and suitable water absorption capacity for wound dressing [119]. In order to provide antibacterial properties, the BA-NFC sponges were soaked, under shaking at 37 °C, in a composites suspension resulted from intercalation of quaternized β-chitin (QC) into interlayer space of organic rectorite (OREC) through electrostatic interactions. The resulting BACNFs/QCRs composites proved their antibacterial property against *E. coli* and *S. aureus* by the obvious appearance of some areas of inhibition compared with BACNFs, which had no antimicrobial capacity. The mouse fibroblast L929 cells, used for in vitro test to evaluate the cytocompatibility of the composites, showed viability higher than 80% with the BACNFs/QCRs concentrations ranging from 5 to 1000 μg/mL, indicating that composites were safe without cytotoxicity. The in vivo animal tests demonstrated the quick hemostasis function of these composites in a rat tail amputation test, dramatically reducing both bleeding time and the amount of bleeding. Moreover, the BACNFs/QCRs could substantially promote collagen synthesis and neovascularization, thus accelerating the wound healing 3 days in advance compared with gauze [119].

#### 3.2.3. BNC-Based Materials in Wound-Dressing Applications

One of the best-known clinical applications of bacterial nanocellulose (BNC) is as topical material in wound healing. Due to its biological properties and high purity, BNC has received more attention as a wound dressing than CNCs and CNFs [16,21,24]. Additionally, the harmless degradation product (i.e., glucose) makes BNC and its composites ideal materials in many wound-dressing applications for bioresorbable purposes [120]. In its indigenous form, bacterial cellulose does not possess any antimicrobial properties. In this sense, in order to improve the competence of bacterial cellulose, it is possible either (i) the chemical modification of cellulose or (ii) the addition of a second component in the network, with biological activities, but also the introduction of antimicrobial agents, metal ions (silver) of various drugs, such as anti-inflammatory, anesthetic, analgesic, etc. [102,121].

Tetracycline hydrochloride (TCH)-loaded BC composites were prepared by immersing BC films into aqueous TCH solution with different concentrations between 0.01 and 0.5 g/L, for 24 h, under gentle stirring. These were examined for drug release, biocompatibility, and antibacterial activity. TCH is a group of broad-spectrum antibiotics used as skin and bone ointments to treat bacterial infections. Thus, the antibacterial activity of TCH-loaded BC composite was investigated using *E. coli*, *S. aureus*, *B. subtilis,* and *C. albicans* as model bacteria and fungus, respectively. The release behaviors of TCH were monitored in 10 mM HEPES (4-(2-hydroxyethyl)-1-piperazineethanesulfonic acid)) buffers at pH 7. After an initial burst release in the first 30 min, the BC-TCH matrix could release the drug in a sustained manner, displaying a steady release for about 10 h. As expected, free-TCH release was faster than TCH-loaded composites, proving the existence of electrostatic interaction between negatively charged BC and positively charged TCH. Antibacterial activity was established after 24 h of incubation by the well-known method of determining the area of inhibition around the samples. It was observed that in the case of the control sample (BC), there are no such areas of inhibition, a fact known from the literature data, namely that BC does not have antibacterial properties. However, in the case of BC composites loaded with TCH, their ability to inhibit bacterial growth has been proven. The best results were obtained for the composite with the highest amount of TCH (0.5 g/L), which reduced *E. coli* growth by 99.98%, *S. aureus* by 100%, *B. subtilis* by 100%, and *C. albicans* by 99.99% after 6 h incubation, respectively. In conclusion, all these beneficial qualities recommend BC-TCH composites as materials with applications as a wound dressing in the controlled release of antibiotics to treat skin wounds [100].

Bacterial nanocelluloses can be used successfully as wound dressings in which various antiseptic solutions can be introduced and subsequently released to the wound site, and among these may be listed: polyoxanide, octenidine, povidone-iodine, etc. [109]. A highly biocompatible system with suitable antiseptic properties was prepared by impregnating BNC with povidone-iodine (PI) and polyhexanide (PHMB) [102]. In vitro tests performed on BNC loaded with PI showed that they did not have side effects on the cells, while contact tests indicated a powerful antibacterial effect on *S. aureus*. These effects were to be expected, given that PI is a complex of polyvinyl pyrrolidone and triiodine ions, which is used as an antiseptic in trauma and orthopedic surgery. During the first 8 h, a fast release could be observed for both antiseptics, which was considerably higher for PHMB (67%) than for PI (46%). After 48 h, PI exhibited a slow-release rate, and the amount of PI (84%) was comparable to PHMB (87%). The release kinetic of PI and PHMB could be described as a combination of diffusion and swelling controlled processes. Regarding biocompatibility assays, the high biocompatibility of BNC loaded with PI against human keratinocytes has been demonstrated. However, by comparison with PHMB-loaded BNC, the antimicrobial activity was significantly lower against *Staphylococcus aureus* [120]. Bacterial nanocellulose was impregnated with octenidine, another antiseptic drug used to obtain an active dressing system. Controlled release studies of octenidine indicated a rapid release in the first 8 h, while in the next 96 h, this was much slower, demonstrating that this system has a time-dependent release profile. Octenidine-loaded BNC proved to have high biocompatibility regarding the human keratinocytes and also an antimicrobial activity against *Staphylococcus aureus*. Moreover, the stability of the BNC-octenidine system for 6 months and the maintenance of biological activity during this period were proven, which indicates the possibility of its use as a dressing for the treatment of infected wounds [101].

Bacterial cellulose-acrylic acid (BC-AA) hydrogels, prepared by electron beam (EB) irradiation, at two irradiation doses of 35 kGy (H35) and 50 kGy (H50), were investigated as wound-healing materials in a rat model. No significant difference in terms of cell viability was observed for the two hydrogels. The percentage of cells viability at 24 and 48 h were more than 85% for both hydrogels. Furthermore, the effects of H35 hydrogels on the healing of partial-thickness burns were evaluated in rats. Macroscopic observations of wound closure, after 1, 3, 7, and 14 days post-injury, revealed clear differences (determined by the sizes of scar grooves) between the H35-treated and controlled untreated groups. At days 1–3, inflammation was observed at the burn site, but no signs of infection, suggesting that the inflammatory phase began in a short time after the injury and lasted for approximately 3 days. On day 7, the untreated control group showed hemorrhagic, scabbed wounds, while in the H35-treated group, no scabbing was observed at the burn sites. The wounds treated with H35 showed nearly complete healing as the treatment approached day 14th, which was not observed in the untreated group. In conclusion, after studying the results, it can be said that BC-AA hydrogels can be considered materials with a high potential to be used for burns due to the fact that they promote wound healing, improving epithelialization and proliferation of fibroblasts [111].

**Figure 6:**
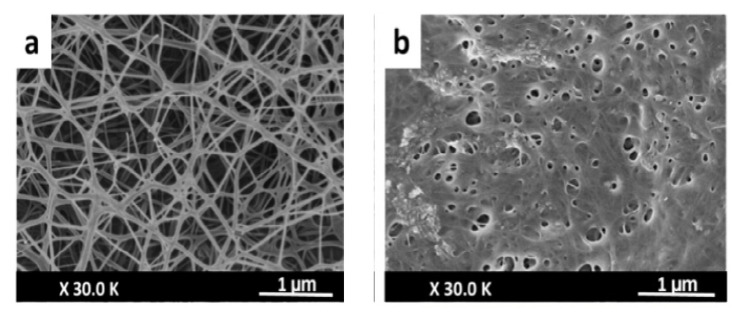
SEM images of (**a**) BC and (**b**) BC-Vac membranes. Adapted with permission from [110]. Elsevier, 2015.

BC membranes loaded with vaccarin were prepared by Qiu et al. 2016 [110] in view of their use in vitro and in vivo as wound-healing materials, due to the fact that vaccarin (Vac), a flavonoid glycoside, has properties to alleviate the oxidative stress damage of endothelial cells in association with skin neovascularization. The morphologies of BC and BC-Vac membranes are shown in Figure 6a,b, when a major change in their structure is observed, from a 3D structure of the BC membrane, with an average diameter of 39.1 ± 9.3 nm, to a 3D nanofibrilar network in the case of the BC-Vac membrane.

**Figure 7:**
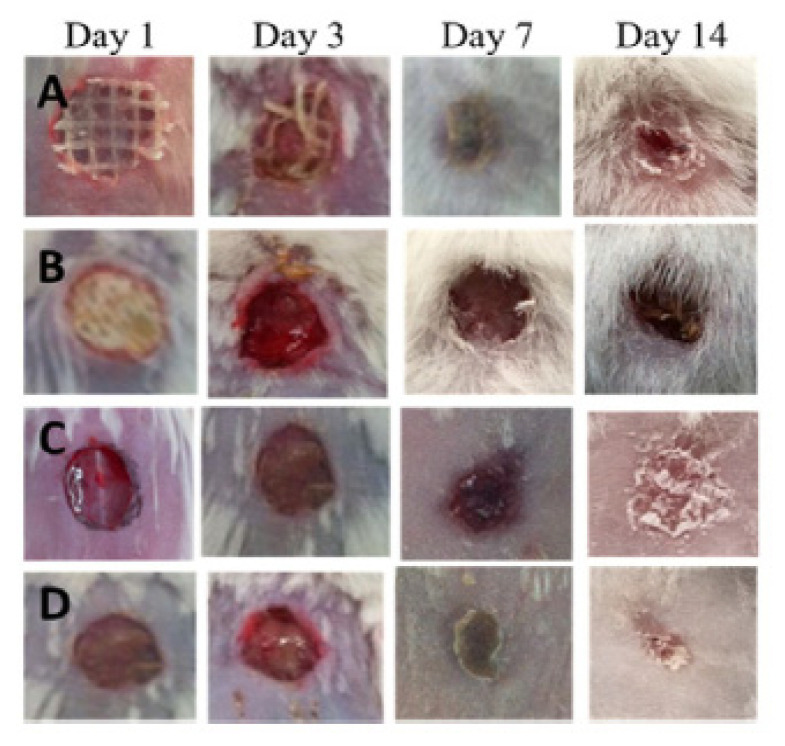
Photographic images on days 1, 3, 7, and 14 of wounds treated with petrolatum gauze (**A**), nano silver dressing (**B**), BC (**C**), and BC-Vac (**D**) membranes. Adapted with permission from [110]. Elsevier, 2015.

Cell viability and cell attachment in vitro showed nontoxicity for both BC and BC-Vac membranes, but improved biocompatibility was exhibited by BC-Vac membranes due to loaded vaccarin that support the endothelial tissue proliferation. Animal studies showed that the wounds covered with BC-Vac healed more rapidly than BC-covered ones. This fact was proved by histological examinations, when a rapid epithelialization of the wounds was observed, only after 14 days, in the case of using BC-Vac membranes (Figure 7). These results show that BC-Vac membranes are very promising materials for wound dressing [110].

Bacterial cellulose can be easily combining with other natural polymers (i.e., collagen, chitosan, alginate) or with inorganic particles (such as ZnO) to improve its properties and resulting in several BC-based nanocomposites [122]. For instance, BC/collagen composites were prepared by immersing wet BC pellicles into collagen solutions. SEM images showed that collagen molecules penetrated into the BC structure and formed multilayers, resulting in an increased Young’s modulus and decreased elongation at the breaking point. These BC/collagen-based composites have been shown to have suitable adhesion and proliferation of 3T3 fibroblast cells, confirming that they have improved cytocompatibility over pure BC. This new material was reported to have the potential for use as a wound-dressing material or artificial skin [79]. In vivo wound-healing potential of BC-zinc oxide (BC-ZnO) nanocomposites was assessed in the burn BALBc mice model. The antimicrobial potential of the nanocomposites was studied against various pathogens that have a role in burns complications, and BC-ZnO nanocomposites successfully inhibited the growth of the tested pathogen (90% for *Escherichia coli*, 87.4% for *Pseudomonas aeruginosa*, 94.3% for *Staphylococcus aureus,* and 90.9% for *Citrobacter freundii*, respectively), indicating that impregnation of ZnO nanoparticles introduced considerable antimicrobial activity into bacterial cellulose. Furthermore, the synthesized composite showed significant (66%) wound healing along with fine tissue regeneration, compared to bacterial cellulose only. On the basis of these findings, it is suggested that these composites of BC could be considered as potential dressing material after clinical evaluation. The worth mentioning characteristic of this dressing is its painless removal from the wound without harming the underlying regenerated tissues [123].

### 3.3. Nanocellulose-Based Materials in Tissue Engineering Applications

Tissue engineering is another area of exciting potential applications for nanocellulose materials [16]. These applications include skin, bone, and cartilage tissue, engineering of blood vessels and soft tissues, repairing congenital heart defects, or ophthalmologic applications [107].

An ideal scaffold for tissue engineering is a material with high porosity, interconnected pores with adequate size, the presence of functional groups for cell adherence, and a high surface-to-volume ratio. Furthermore, tissue engineering scaffolds should possess suitable mechanical properties and should be nontoxic for cells. All these physicochemical properties are essential for cells growth and proliferation, vascularization, and nutrient transfer [124,125].

Cellulose-based materials have been shown to have great biomedical potential in the construction of ECM-mimicking scaffolds owing to their intrinsic characteristics, such as biocompatibility, lack of cytotoxicity, tunable 3D architecture and porous microstructures, and desired mechanical properties, although in vivo degradability still remains under discussion due to lack of the relevant enzyme in the human body [48,62,115,126]. In particular, the use of cellulose nanoparticles to improve the performance of other polymers used as scaffolds is another topic of interest for the researchers. Nanocellulose is considered one of the most promising nano-reinforcement materials of composites due to its nanoscale size, high aspect ratio, and excellent mechanical strength. Compared to other nanofillers, the incorporation of nanocellulose from natural sources could greatly improve the mechanical property of the polymer matrix, but without the sacrifice of its biocompatibility and biodegradability. Last but not least, the presence of surface hydroxyl groups in nanocellulose not only makes the chemical modification easy but also enhances the hydrophilicity of the materials, which is beneficial to the growth and proliferation of cells [127,128]. Nanocellulose-based materials applications in tissue engineering are vast and varied, and Table 3 presents some examples, from the multitude of applications in this field, for each type of nanocellulosic material.

#### 3.3.1. Nanocellulose-Based Materials as Scaffolds in Skin Tissue Engineering

The first investigations regarding the use of CNCs as a candidate for tissue engineering scaffolds were carried out about 10 years ago by Dugan and coworkers [149,150]. In the first investigation [149], high-aspect-ratio cellulose nanowhiskers (CNWs) derived from the tunicate *Halocynthia roretzi* were deposited onto glass substrates by a simple spin-coating technique. Myoblasts were oriented and differentiated along CNW surfaces when they produced radial models of myotubes that followed the radial pattern of CNWs, thus demonstrating for the first time the possibility of using CNWs in tissue engineering applications. In the second study [150], CNCs extracted from the marine invertebrate *Ascidiella aspersa* were oriented on glass coverslips with a similar spin-coating method. CNCs with nanoscale dimensions were shown to induce a similar guidance response in C2C12 skeletal muscle myoblasts, but in addition promoted the degree of myoblast fusion, terminal differentiation, and template deposition of an oriented fibrillar extracellular matrix.

The design of some skin tissue engineering scaffolds and in vitro testing of the response of human skin cells to them was performed by incorporating cellulose nanocrystals (CNC) in polyvinyl alcohol (PVA) hydrogels [131]. PVA is known for its biodegradable and biocompatible properties, and the 3D structure of its hydrogels has attracted interest in mimicking extracellular matrices for tissue engineering [151]. Clearly, the porous structure of PVA hydrogels was affected by CNCs addition, so all PVA/CNC nanocomposites have a highly porous structure with irregular and interconnected pores with an approximate size range of 37–164 μm. Besides porosity, other properties such as mechanical, thermal, and swelling were significantly improved by CNC addition. For instance, the PVA/NCC scaffolds with 4 and 6 wt.% NCC exhibited higher equilibrium swelling ratios of 853% and 850%, respectively. The in vitro evaluation of the cytotoxicity and cell culture of PVA/CNC with 10% CNC revealed a noncytotoxic effect of biomaterial and a strong attachment, proliferation, and penetration of human fibroblast skin cells on the scaffold was observed [131].

Hydrogels of gelatin (GA), hyaluronic acid (HA), and cellulose nanocrystals (CNC) (GA-HA-CNC) were prepared by freeze-drying method and tested regarding the capacity to promote the attachment, growth, and proliferation of fibroblasts. The three components with different characteristics will have consequently different functions: GA provides a similar extracellular matrix environment for cells to adhere, grow and proliferate [23,139]; HA, with high water-binding properties, participates in tissue hydration and promotes trackless wound healing [152,153]; CNC serve as reinforcing material, significantly enhancing the property of the hydrogels and playing a vital role in the rheology and swelling results. The GA-HA-CNC hydrogels have a smooth surface with a pore size of 80–120 μm, which can provide sufficient volume and surface for cell growth. NIH-3T3 cells were used to evaluate the ability of the GA-HA-CNC hydrogels to support cell viability and proliferation. NIH-3T3 cells were cultured in GA, GA-CNC, HA, HA-CNC, GA-HA, and GA-HA-CNC hydrogels, respectively. Cell viability in GA-HA-CNC hydrogels, determined at 1, 4, and 7 days, were significantly higher than other hydrogels, the phenomenon being attributed to the cumulative effects of the three components that promoted the diffusion of tissue fluid and nutrients and the similar extracellular matrix environment that prevented the foreign body response. Consequently, it can be stated that the GA-HA-CNC hydrogel has a high potential in use for repairing skin wounds [132].

**Figure 8:**
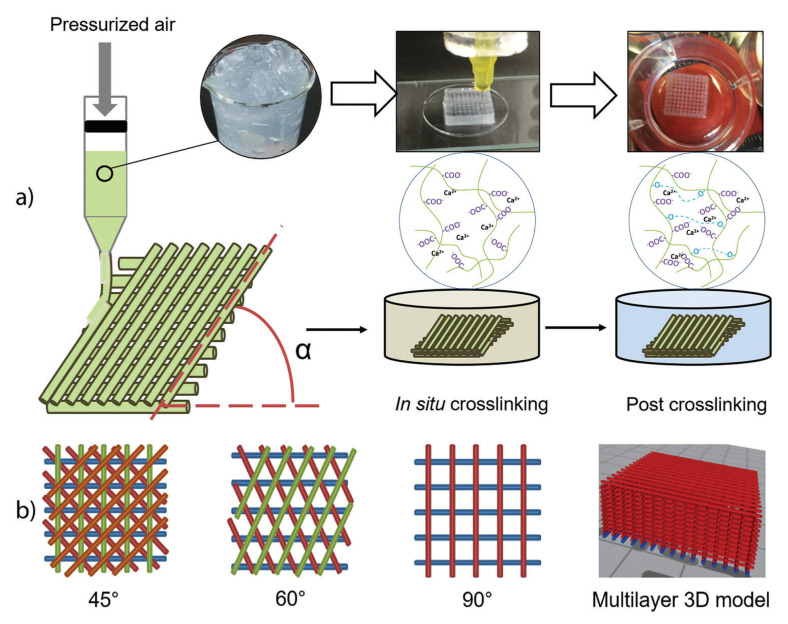
Illustration of 3D printing of CNF hydrogels: (**a**) schematic illustration of the printing process and the two-step crosslinking strategy; (**b**) printing patterns, top view of 45°, 60°, and 90° patterns, and 3D view of the 90° scaffold model. Adapted with permission from the [115]. The Royal Society of Chemistry, 2018.

CNFs turned out to be favorable for cell culture due to their small fiber diameter and large surface-to-volume ratio. Moreover, the CNF hydrogels have a controllable porosity that is necessary for efficient cellular infiltration and nutrient diffusion. However, a disadvantage can be mentioned, namely the lack of the adhesive sites necessary for cell signaling and migration, so further modification and functionalization are required to use CNFs as culture platforms [63]. Three-dimensionally printed CNFs hydrogel scaffolds were prepared by double crosslinking, for the first time, by Xu and coworkers [115]. First, in situ Ca^2+^ crosslinking was made, followed by post-printing chemical crosslinking with 1,4-butanediol diglycidyl ether (BDDE) (Figure 8). It turned out that this double crosslinking allowed an increase in the mechanical strength of the scaffolding in the range of 3–8 kPa. Cell survival on 3D-CNF scaffolds was tested with human dermal fibroblasts (HDF) isolated from the human foreskin and involved in wound healing, and cell adhesion capacity was measured after 12 h of cell incubation. In comparison with 2D control samples, cells adhered slightly less on the 3D-printing matrix, with the observation that the matrix thickness influences the cell adhesion, i.e., 3 mm samples having a higher percentage of cell attachment (84%–86.5%) than 2 mm samples (78.25%–79.25%). This could be due to the effect of the larger surface areas of the former for cells to adhere. A remarkable advantage of 3D CNF materials was that they support cell proliferation after longer incubation periods. After 3 days post cell seeding, there are 2–4 times more HDF cells on 3D CNF samples as compared to 2D control samples. In conclusion, the cell tests confirmed that the 3D-printed CNF hydrogel scaffolds are nontoxic to fibroblast cells [115].

Highly transparent NFC-chitosan (CS) nanocomposites were prepared by solvent casting method in order to obtain an artificial skin with mechanical properties equivalent to native skin. The presence of NFC nanoparticles in the chitosan matrix suppressed the water uptake of CS and increased the transparency and mechanical properties of NFC-CS nanocomposites. The mechanical strength (yield strength and Young’s modulus) of nanocomposites, in dry conditions, increased with increasing of NFC content due to the NFC reinforcement effect. It turned out that the improvements of mechanical strength for wet samples were larger than those for dry samples: ultimate strength was improved by 25% for dry samples and 12 times for wet samples, respectively, compared to dry and wet CS samples. In conclusion, the mechanical behavior of wet NFC-CS nanocomposites matched very well those of human skin, and their prominent properties could be exploited for applications in artificial skin and wound dressings [30].

A one-step freeze-drying technique was used to fabricate a bilayer scaffold from cellulose nanofibers and poly(vinyl) alcohol. The bilayer scaffold mimics the two layers of the skin, the dermis, and epidermis, respectively. The scaffolds’ porosity and their mechanical and swelling properties were varied by controlling the concentration of polymers. CNF/PVA scaffolds were evaluated regarding their potential use in tissue engineering for skin repair. SEM analysis confirmed the existence of interconnected pores in the scaffolds, with two different pore sizes for the epidermis and dermis, 19.72 ± 3.6 and 90.71 ± 2.4 μm, respectively. The scaffolds made from polymers with lower concentrations (N1) had higher porosity than those made with higher polymer concentration (N2), with larger pore size, increased water uptake, and decreased mechanical strength. Scaffold biocompatibility was measured on the 1st and 3rd day by MTT assay on the fibroblasts cells and keratinocytes cultured on both layers of CNF/PVA scaffolds as well as the 96-well plates as the control group. All samples showed suitable cell viability (more than 80%), but the cell viability on N1 and N2 scaffolds were better than PVA scaffolds in both culture periods. This may be the result of nanocellulose influence with its nanostructure that can mimic the extracellular matrix and hence enhance cell attachment and proliferation. All results proved that these bilayer scaffolds could meet the requirements necessary to be used in tissue engineering for skin repair [129].

Collagen (COL) is one of the major components of the extracellular matrix, recognized for its excellent biocompatibility and biodegradability, but also for its low bioactivity, which restricts its practical application [144]. To overcome this limitation, Wen and coworkers [142] used dialdehyde bacterial cellulose (DBC) to composite collagen peptide (Col-p) via covalent bonds between the aldehyde group of DBC and amino functional groups of Col-p. The nanocomposites DBC/Col-p were prepared by immersing DBC with various degrees of oxidation into Col-p solution and explored for the release of collagen peptides compared to BC/Col-p. In vitro desorption measurements of Col-p suggested a controlled release mechanism from the DBC/Col-p: above 85% of Col-p was fully released from BC/Col-p within 12 h, while 45–60% Col-p was released from DBC/Col-p; after 60 h, more than 92% Col-p was released from the BC/Col-p, while about 68–87% Col-p was released from the DBC/Col-p. The biocompatibility of nanocomposites was evaluated by culturing with fibroblasts from neonatal rats on the 1st, 4th, 7th, and 10th days. The cells number increased obviously starting on the 4th day and continued to grow on the 7th day, in well-spread ways, when the confluence also occurred. In conclusion, the DBC/Col-p nanocomposites showed suitable fibroblast cell adhesion and proliferation and proved to be a promising material for tissue engineering and regeneration [142].

#### 3.3.2. Nanocellulose-Based Materials as Scaffolds in Bone Tissue Engineering

Bone is a matrix comprised largely of collagen mineralized by hydroxyapatite [78]. Its tissue is made up of different components such as osteoblasts, osteocytes, and osteoclasts [154]. For successful bone regeneration, an ideal bone scaffold must present three main properties: osteoconduction, osteoinduction, and osseointegration. *Osteoconduction* refers to the ability of the scaffold to promote the growth of new tissue on the external and internal pores of the implant [155]; *osteoinduction* describes the capacity of the scaffold to attract immature cells to a healing site and stimulating them to develop into bone-forming cells; and *osseointegration* is a time-dependent healing process that defines the anchoring of an implant through direct contact with bone, a critical process in implant stability [156].

As a scaffold in bone tissue engineering, crystalline nanocellulose is used either as biomaterials with enhanced mechanical and biocompatibility properties or as reinforcing agents and nanofillers for other synthetic or natural polymers [126].

Binary-component composite hydrogels were prepared, by covalent crosslinking, from polyacrylamide (PAAm) networks reinforced with cellulose nanocrystal (CNC) skeleton. There was a synergistic effect between the hardness of the CNC skeleton and the elasticity of the PAAm network, which lead to composites with excellent mechanical properties and excellent fatigue resistance, the CNC skeleton contributing to a simultaneous improvement in hardness and elasticity of almost 500% compared with PAAm hydrogels only. This is a great strategy to use the CNC skeleton as a reinforcing template, which offers the possibilities to obtain composites with exceptional mechanical properties important for bone scaffolds tissue applications where high strength is required [136].

The electrospinning technique was used to produce nanocomposite scaffolds from poly(lactic acid) (PLA) and poly(ethylene glycol)-grafted cellulose nanocrystals (CNCs-g-PEG) as a reinforcing filler. By grafting with PEG, improved dispersion of cellulose nanocrystals in the PLA matrix was achieved. The PLA/CNCg-PEG nanocomposites were characterized from a morphological, thermal, mechanical, and wettability point of view, as well as in terms of biocompatibility with human mesenchymal stem cells (hMSCs). The reinforcing filler addition, either in the form of CNC or CNC-g-PEG, has a negative influence on the average diameter and the glass transition and cold crystallization temperature; while it has a positive effect on the tensile strength of the electrospun nanofibers, 5% loading seemed to be the optimal content level. Regarding the biocompatibility with hMSCs cells, both the PLA/CNC and PLA/CNC-gPEG composites exhibited suitable biocompatibility, being nontoxic to hMSCs and capable of supporting cell attachment and growth. However, PLA/CNC-g-PEG (5%) scaffold with enhanced mechanical strength and decreased diameter of nanofibers exhibited improved cell viability and proliferation cell count, which shows promises for its potential applications in bone tissue engineering [134].

The cellulose nanofibrils-reinforced gelatin (GEL/CNF) composites with variable composition (6/0, 5/1, 3/3, 1/5 volume ratios) were analyzed regarding their potential as bone TE scaffolds. Their morphological, mechanical, and physicochemical properties, as well as their bioactivity and biological response by means of the osteogenic environment of mesenchymal stem cells (MSCs), were evaluated. The scaffolds possess a suitable microstructure with osteoid-like compressive strength and elasticity, being capable of evoking calcium deposition. In addition, the introduction of biomimetic phosphonate (ApA) moiety to CNFs intensified even more the deposition of HAp-like crystals on the scaffolds, generating an osteointegrative bone-scaffold interface. None of the materials caused any cytotoxic effect to the MSCs, thus enabling cell viability, proliferation, and mineralization for bone tissue engineering. On the other hand, the MTT staining on all GEL/CNF scaffolds indicated that a significant number of viable cells were present on the surface as well as in the vicinity of all tested scaffolds, even after five weeks of MSC cultivation. The results proved that the GEL/CNF scaffolds may be identified as promising candidates for bone tissue engineering in non-load bearing applications, such as sinus augmentation or as extraction grafts, opening new options for cell-based therapies by the usage of nanocellulose [130].

The use of bacterial nanocellulose (BNC) as a potential bone tissue scaffold is based on its morphological similarities with collagen, which could act as a matrix for providing maximum integration with cells and body fluids [78]. Therefore, many research groups have prepared suitable composites having BNC and collagen in their composition to mimic the basic chemical composition of natural bone [143]. A homogeneous composite scaffold was fabricated from alginate (ALG), bacterial cellulose nanocrystals (BNCs), and collagen (COL) using calcium carbonate in the presence of D-glucono-δ-lactone (CaCO_3_–GDL complex) as the gelling system. In vitro cytocompatibility was evaluated using the osteoblastic MC3T3-E1 cells and human adipose-derived mesenchymal stem (hAMS) cells. The results, after 2 and 5 days of incubation, showed that both MC3T3-E1 and hams cells were viable and proliferated well on all three scaffolds (ALG, ALG-COL, and ALG/BCNs/COL, respectively), with some differences related to composition and architecture of the scaffolds: ALG/COL and ALG/BCNs/COL exhibited suitable proliferation, significantly higher than ALG and control. The variation was attributed to the poor cell attachment of ALG, suitable cellular affinity of COL, and suitable biocompatibility of BCNs, which improved the final biological behavior of the composite scaffold. Consequently, ALG/BCNs/COL possessed suitable 3D architecture, higher porosity, enhanced compressive strength, appropriate swelling, desired biodegradation rate, and suitable cytocompatibility, making them an ideal scaffold for tissue engineering [140].

The properties of nanocomposites based on COL, NCBs, and carbonate apatite (Ap) in association with osteogenic growth peptide (OGP) and its C-terminal pentapeptide [OGP(10-14)], were evaluated when demonstrated in vitro bioactivity and osteoinductive properties [157] showed that OGP peptides conferred an osteoinductive property to the BNC membrane by influencing the osteogenic proliferation and favoring the mineralization process. The (BC-COL)-Ap composites did not exhibit cytotoxicity, genotoxicity, or mutagenicity effects. Furthermore, in vitro assays showed synergism between collagen, apatite, and OGP peptides, which consequently induced early development of osteoblastic phenotype compared to BC-Ap nanocomposite. These results suggest that the (BC-COL)-Ap associated with OGP peptides proved to be a suitable candidate for bone tissue engineering applications [144].

Bacterial nanocellulose-gelatin/hydroxyapatite composite with double-network architecture was prepared by immersing the hydroxyapatite-coated BNC (BNC/HAp) in gelatin (GEL) solution at 45 °C for 7 days [158]. The SEM images showed that HAp particles, with 300–400 nm in diameter, were located at the nodules of the BNC network, while GEL molecules filled the porous structures of the BC network. In a previous study [159], BNC/HAp composite proved to be effective in bone regeneration but has poor mechanical strength limiting its widespread applications in bone tissue engineering. Thus, by immersing the BNC/HAp in gelatin, the mechanical strength has been significantly improved both in the wet and dry state compared with BC/HAp hydrogels (i.e., in wet state elastic modulus increase from 0.12 to 0.28 MPa, while in the dry state increase from 48 to 177 MPa). The proliferation of rBMSCs culture on the BC, BC/HAp, BC/GEL, and BC-GEL/HAp and tissue culture plastic (control group) was followed on days 1, 4, and 7. The rBMSCs grew continuously during the observation period, but from day 4, increased proliferative capacity was observed for BC/HAp and BC-GEL/HAp scaffolds. The same behavior was observed in rBMSCs differentiation culture. These results can be attributed to the architecture of the BC/HAp and BC-GEL/HAp scaffolds, which possess the urchin-like HAp nanoparticles with high surface area located at the nodules of the BC network and could greatly enhance the surface roughness, facilitating the adhesion, proliferation, and differentiation of rBMSCs [158].

**Figure 9:**
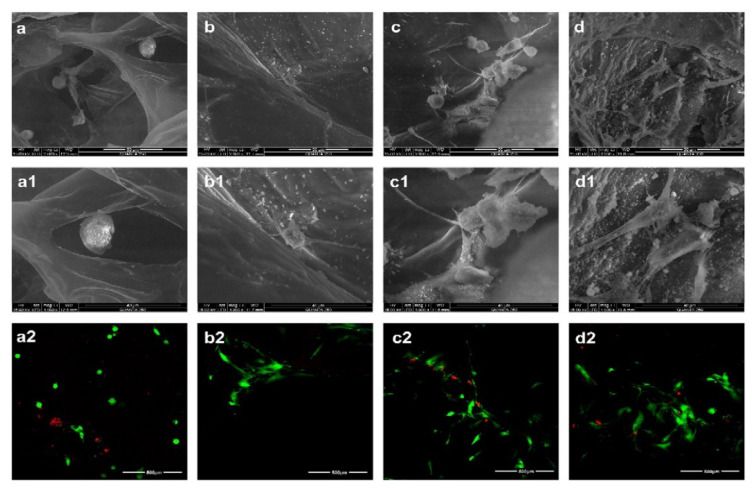
Morphology and viability of hBMSCs seeded in BC (**a**–**a2**), BC/Gel (**b**–**b2**), BC/PA/Gel (**c**–**c2**), and BC/PA/Gel/HAp (**d**–**d2**) scaffolds for 1 day by SEM and 3 days by live/dead staining. Original magnification: 2000× (**a**–**d**), 4000× (**a1**–**d1**), and 200× (**a2**–**d2**). Live and dead cells were stained with green and red, respectively. Reproduced with permission from [143]. Elsevier, 2017.

Nanostructured macroporous BNC scaffolds, prepared via freeze-drying technique, were immersed in gelatin (Gel), crosslinked with procyanidin (PA), and coated with hydroxyapatite (HAp). Thus, four scaffold types resulted (BNC, BNC/Gel, BNC/PA/Gel, and BNC/PA/Gel/Hap), which were tested for their biocompatibility and osteoinductivity on the human bone marrow stromal cells (hBMSCs). The adhesion and viability of hBMSCs were investigated after seeding on the different scaffolds for 1 and 3 days, respectively. As the SEM pictures and the live/dead staining images showed (Figure 9), significant differences are observed in hBMSCs behavior: in BNC scaffolds, the hBMSCs were distributed separately with round and unexpanded shape; in the BNC/Gel scaffolds, the cells extended pseudopodia of filament, but they are still individualized; in the BNC/PA/Gel scaffolds, the cells forms aggregated and clustered, indicating the cells proliferation; in the BNC/PA/Gel/HAp scaffolds, the adherent cells markedly increased and connected with each other, suggesting suitable intercellular communications. Thus, the best adhesion, viability, proliferation, and osteogenic differentiation of the hBMSCs appeared on the BNC/PA/Gel/HAp scaffold, followed by BNC/PA/Gel, BNC/Gel, and BNC [143].

In vivo tests, after implantation of the scaffolds into muscle pouches of nude mice, showed clearly visible fibers in all scaffolds, suggesting poor degradation of BNC. As well as the results in vitro, in vivo investigations showed the same order of new bone formation, from high to low was: BNC/PA/Gel/HAp, BNC/PA/Gel, BNC/Gel, and BNC scaffolds. In conclusion, the modification of BNC by gelatin and hydroxyapatite coating greatly promoted the biocompatibility and osteoinductivity of pure BNC scaffolds, which can be used as a potential orthopedic implant for bone repair applications [143].

**Figure 10:**
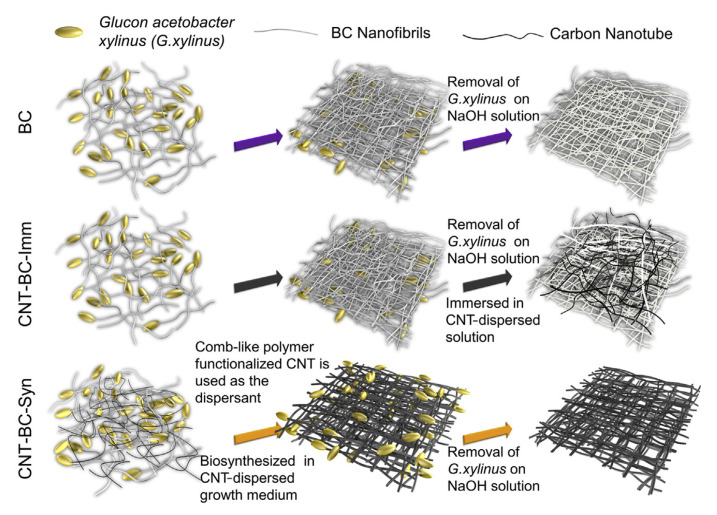
Schematic illustration of the 3D scaffold fabrication processes of BC, CNTs-BC-Imm, and CNTs-BC-Syn. Adapted with permission from [145]. Elsevier, 2015.

Carbon nanotubes (CNTs) are materials with high potential application in biomedicine, especially in regeneration medicine, due to their chemical composition, topography, roughness, and stiffness [160]. However, in vivo applications of CNTs have been hampered by difficulties associated with the CNTs’ 3D scaffolds fabrication. Park and coworkers devised a new method for biosynthesis of CNTs-based 3D scaffolds by in situ hybridizing CNTs with bacterial cellulose (BC), more exactly by culturing BC-synthesizing *Gluconacetobacter xylinus* bacteria in a medium containing CNTs. In addition, an amphiphilic comb-like polymer (APCLP) was adsorbed on CNTs to obtain a homogeneous distribution of CNTs throughout the 3D microporous structure of BC (Figure 10) [145].

The BC-carbon nanotubes composites were tested as bone regeneration scaffolds through mesenchymal stem cells differentiation. The APCLP-adsorbed CNTs-BC hybrid scaffolds (CNTs-BC-Syn) showed excellent osteoconductivity and osteoinductivity, leading to high bone regeneration efficacy, compared with CNTs-BC-Imm scaffolds obtained by simple immersion of 3D-BC scaffolds in CNTs solution. The nanoscale CNTs-BC-Syn scaffold, with a pore size of 85 μm, is favorable for cell attachment, proliferation, alkaline phosphatase synthesis, and extracellular calcium deposition, which are essential processes in bone regeneration. Moreover, CNTs-BC-Syn showed mechanical strength appropriate for osteogenic differentiation, and the hydrogel-like BC layers surrounding CNTs may act as a reservoir for accommodating various growth factors, which in turn can enhance the cell differentiation to facilitate bone regeneration [145].

#### 3.3.3. Nanocellulose-Based Materials as Scaffolds in Vascular and Cardiac Tissue Engineering

Because of the limited supply of blood vessels from our own body and possible severe rejection effects induced by allograft, there is a large amount of need to use artificial vessels as a substitute [161]. Intravascular blood flow can produce a shear effect in varying degrees; thus, vascular scaffolds, in addition to excellent biocompatibility and controllable biodegradability, should have tunable strength to meet the mechanical requirements of blood vessels at different sites [162]. Usually, biocompatible natural polymers (collagen, hyaluronic acid) lack mechanical strength [163], while synthetic polymers (expanded polytetrafluoroethylene, polyethylene terephthalate, poly-L-lactic acid, poly(glycolic acid)) lack adequate cell affinity [130,164] and sometimes contribute to the formation of thrombi and intimal thickening [161].

The native bacterial cellulose can be considered a promising material in the construction of artificial blood vessels due to its specific properties, which are superior to many synthetic materials [120,165]. These may include: (i) through specific techniques, BNC can be processed into tubes of various lengths and diameters, with suitable blood compatibility, demonstrated by in vitro and in vivo tests; (ii) blood compatibility can be increased by modification of the interior surface of BNC scaffolds; (iii) to improve the cell ingrowth an option is a modification the porosity of the BNC scaffolds; while (iv) the mechanical properties are similar to those of blood vessels [78].

Bacterial nanocellulose, biosynthesized with the help of *Gluconacetobacter xylinum*, was used as a material to obtain tubular scaffolds with morphological and mechanical properties similar to native blood vessels [161]. BC tubes with a length of 100 mm, a thickness of 1 mm, and an outer diameter of 4–6 mm and were prepared using a tubular template material made from polydimethylsiloxane (PDMS). PDMS is known as a synthetic macromolecule derived from organosilicon compounds with high transparency, oxygen permeability, inertness, and nontoxicity [166]. The SEM analysis, tensile testing, and thermal analysis showed that BC tubes have nanofiber architecture, suitable mechanical properties, and thermal stability. For instance, the deformation behavior of BC tubes is better than the porcine carotid artery: Young’s modulus for 4 mm tubes was 3.94 ± 0.12 MPa, and for 6 mm tubes was 3.30 ± 0.19 MPa, while for porcine carotid artery was reported to be around 1.04 Mpa. BC tubes are also transparent, elastic, and mimic real blood vessels. Evaluation of cytotoxicity and cell compatibility suggests that BC tubes have no toxic or side effects on vessel-related cells cultured on their surface, which demonstrated that the surface of BC tubes was beneficial for cell attachment, proliferation, and ingrowth. Hence, BC tubes appear to be appropriate for applying in vessel transplantation and reconstructive surgery [161].

Composites based on bacterial cellulose were fabricated by immersing the never-dried BC sheets in the fibrin solution with different concentrations (26–33 wt.%) and crosslinked with glutaraldehyde. The BC/fibrin composites borrow both the properties of BNC (i.e., superior strength and stability in the wet state) and fibrin (i.e., elasticity). As expected, BNC/fibrin composites display significantly higher strength (1.29–1.32 vs. 0.95 MPa) and higher strain at break (0.33 vs. 0.19) over native BNC. These results demonstrated that the BNC/fibrin composites have properties comparable to those of native blood vessels, such as the bovine coronary artery [78].

A porous scaffold based on collagen, cellulose nanocrystals, and gelatin microspheres (GMs) was reported by Li and coworkers [133] by incorporation of GMs into collagen/CNCs scaffold. The scaffold was produced as a platform for a basic fibroblast growth factor (bFGF) sustained release and formation of blood vessels (angiogenesis). Different amounts of CNC were added to collagen solution in 0.5 M acetic acid corresponding to 0, 1, 3, 5, 7, and 10 wt.%, respectively, of the collagen mass. The amount of bFGF loaded into each scaffold was 10 ng. The thermal degradation behaviors showed that a composite scaffold with 5 wt% CNCs resulted in maximum thermal stability and had a degree of swelling sufficient to absorb a large amount of exudate in the early stages of wound healing. The study of release kinetics of bFGF from collagen/CNCs/GMs scaffolds showed a fast release (24%) in the first 2 days, and by day 14th, the cumulative release reached 70%; while bFGF delivered directly almost disappeared at 7 days. The cytotoxicity tests consisted of human umbilical vein endothelial cells incubated into the scaffolds for 7 days. The scaffolds loaded with bFGF were implanted subcutaneously in Sprague–Dawley rats for in vivo angiogenesis studying when the formation of high densities of new blood vessels in the scaffold was observed. This study suggests a rapid and effective method of promoting angiogenesis for wound repair, with great clinical potential for engineering tissue constructs to induce angiogenesis [133].

#### 3.3.4. Nanocellulose-Based Materials as Scaffolds in Cartilage Tissue Engineering

The potential use of nanocellulose materials in cartilage TE was evaluated by Nimeskern and coworkers [146], who investigate the bacterial nanocellulose (BNC) as biocompatible and non-degradable material to promote chondrocyte adhesion and proliferation in ear cartilage tissue engineering (TE). The BNC scaffolds were prepared with cellulose content between 2.5% and 15%, and their stress relaxation indentations were compared with those of human ear cartilages. The instantaneous (E_in_) and equilibrium modulus (E_eq_), and also maximum stress σ_max_ for BNC samples correlate well with cellulose content, and BNC with 14% cellulose content reached values equivalent to ear cartilage (i.e., E_eq_ = 2.4 ± 0.4 MPa for BNC, E_eq_ = 3.3 ± 1.3 MPa for ear cartilage, respectively). In conclusion, BNC scaffolds have the capability to reach mechanical properties of ear cartilage, and additionally, can be manufactured in shapes suitable for patient-specific auricular shape [146].

Bilayer scaffolds-based bacterial nanocellulose was obtained by joining one compact network layer of BNC with a porous composite layer of BNC and alginate. The scaffolds were seeded with human nasoseptal chondrocytes (hNCs) combined with human mononuclear cells (hMNC) from bone marrow [147]. The alginate hydrogel maintains a chondrogenic phenotype of human chondrocytes and stimulates neocartilage formation, reasons for the selection of these in the preparation of the porous composite layer [167,168]. The BNC layer confers suitable mechanical characteristics to the scaffold, while the composite layer has interconnected high porosity with a mean pore size of 50 ± 25 mm. Two-layer scaffolds were tested for 6 weeks to determine their viability, when they were found to be noncytotoxic, with cell viability of 98% and uniform distribution of cells on the entire porous layer, even in the center of the scaffold, a critical region in 3D static culture due to the limited intake of nutrients and oxygen. In vivo studies, conducted by implantation of the scaffolds in nude mice for 8 weeks, demonstrated the deposition of cartilage matrix components (pro-theoglycan and type II COL). In conclusion, it has been shown that newly formed scaffolds have suitable mechanical stability and maintain their structural integrity over a long period of time, in addition to giving adequate porosity to cell growth and neocartilage formation [147].

Bacterial nanocellulose-chitosan scaffolds were prepared by a vectorially aligned freeze-drying technique by incorporation of chitosan inside the BC network. The use of this technique leads to vectorial alignment of the BNC nanofibrils along the freezing direction. It was observed that by varying the concentration of chitosan (1, 1.5, and 2 wt.%), the pores size of the scaffold and the distance between the channels can be changed. Moreover, the incorporation of chitosan in the scaffold allows the realization of a dense network, with suitable structural integrity and excellent recovery of shape after removal of the load, unique properties that can be adapted for the regeneration of cartilage (cartilaginous tissue) [148].

Three-dimensional (3D) bioprinting, one of the most promising and advanced technologies to fabricate scaffolds with specific features, was found to be suitable for obtaining 3D printing scaffolds for cartilage TE [169]. Three-dimensional printing enables the creation of individual, tailor-made scaffolds to provide desired architectures, and furthermore, integration with biological cues to target cell proliferation in a controlled manner [115]. Although there are numerous biocompatible polymers reported in literature, few are fulfilling the considerable requirements for ink formulations and subsequent production of 3D scaffolds [170]. Hydrogels have attracted attention as suitable materials for bioinks because of their high water content, which gives them a structural similarity to the extracellular matrix. However, in order to be suitable for 3D bioprinting, the hydrogel viscosity must be high enough for the hydrogel to retain its shape during printing, and also, the hydrogel must allow crosslinking to be retained in the 3D structure after printing [171].

Compared with the other biopolymers, nanocelluloses stand out in the field of bioink formulation owing to their high mechanical strength as well as the structural similarity mimicking natural ECM [172]. The nanoscale of cellulose makes it a versatile and sustainable bio-nanomaterial that holds great potential for the fabrication of 3D constructs. Nanocellulose can be used either as a main component or as a reinforcing agent in different polymer-based composite bioinks [82,173]. A bioink composite formula based on nanofibrillated cellulose (NFC) and alginate was prepared for 3D bioprinting of soft tissues. The bioink formula combines the rheological properties of NFC with the alginate fast ability to cross-link, thus proving an excellent printability at room temperature and low pressure. The 3D-printed bioink structures representing 3D cartilage tissue were tested for cytotoxicity and cell viability with hNC (human nasoseptal chondrocytes). The bioinks proved to be biocompatible and suitable materials for cell culture statements supported by results obtained for the viability of hNC bioprinted in NFC-based bioink, which is 73% after 1 day and 86% after 7 days. Thus, it was demonstrated that NFC/alginate bioinks are adequate hydrogels, which can be used with living cells in 3D bioprinting for the development of cartilage tissue [171].

#### 3.3.5. Nanocellulose-Based Materials as Scaffolds in Soft Tissue Engineering

Polyvinyl alcohol (PVA)-based hydrogels were reinforced with cellulose nanocrystals (CNCs) to obtain CNCs–PVA hydrogels using the freeze-thawing technique. Due to the reinforcement with CNCs, the PVA hydrogel characteristics change considerably. It was found that the mechanical and viscoelastic properties of the hydrogel are dependent on the solvent used in the synthesis (DMSO:water ratios) and can be altered with CNC addition. The microscopy results show that the hydrogels possess an open macroporous structure in their native state. The viscoelastic properties, established by dynamic viscoelastic analysis and the mechanical ones, determined by axial compression tests, proved that the addition of CNC allowed the strengthening of the hydrogel’s network. In addition, the presence of PVA permitted the realization of hybrid hydrogels with a high capacity to retain water, is flexible, and has the ability to be poured in different forms. All properties of these hydrogels provide them the possibility to be used in soft tissue applications [135].

Soft tissue engineering finds applications also in ophthalmic tissues regeneration, but in these applications, specific features are important for materials, besides biocompatibility, nontoxicity, and non-immunogenic response. Depending on the application, the materials should have excellent optical properties, enough oxygen permeability, be able to hold high water content, be relatively soft but strong enough mechanically but be sufficiently strong enough to be sutured for implanting [63,138].

**Figure 11:**
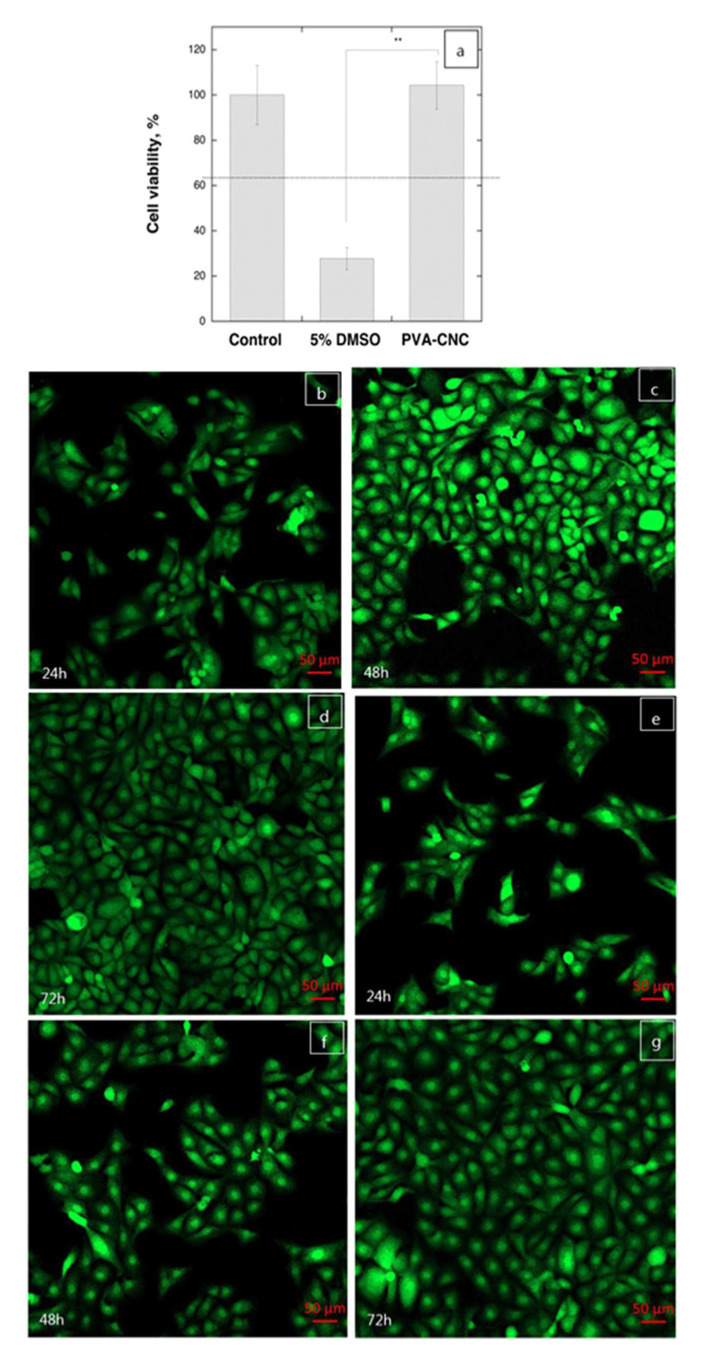
Biocompatibility study: (**a**) cell viability of HCE-2 cells cultured in extract medium of CNC-PVA/DMSO; the data are expressed as percentage of the negative control (tissue culture plate extract medium) and represent the mean ± SEM (** *p* < 0.01). (**a**–**c**) Confocal microscopy images of GFP-labeled HCE-2 cells cultured on CNC-PVA hydrogels: (**b**) growth after 24 h, (**c**) growth after 48 h, (**d**) growth after 72 h. (**e**–**g**) Confocal microscopy images of GFP-labeled HCE-2 cells cultured on TCP: (**e**) growth after 24 h, (**f**) growth after 48 h, (**g**) growth after 72 h. Reproduced with permission from [138]. American Chemical Society, 2016.

Nanocellulose is considered an ideal material for ophthalmic TE due to its large number of hydrogen bonds, prominent water absorption, mechanical strength, suitable compatibility, and optical transparency [162,174,175,176,177]. TEMPO-CNC reinforced polyvinyl alcohol (PVA) hydrogels were reported for ophthalmic applications by Tummala et al. [138]. PVA is a synthetic material with high transparency, suitable wettability, excellent biocompatibility, and non-adherent properties related to the abundance of hydroxyl groups along its chains [135]. Additionally, nanocellulose produced from TEMPO-oxidized cellulose is transparent, has a high water-holding capacity, and is shown to be cytocompatible [178]. The contact lenses obtained from CNC-PVA hydrogels present 95% transparency in the visible range and moderate UV-absorption properties and also showed rubber-like mechanical properties, being soft and elastic. CNC-PVA hydrogels have been shown to be nontoxic and to have excellent biocompatibility, established by the indirect cytocompatibility test when it was shown that the viability of corneal epithelial cells (HCE-2) cultured in hydrogel extractive medium was above the limit of 70% and much higher than the positive control (5% DMSO in culture medium) (*p* < 0.01) (Figure 11). Moreover, it has been shown that HCE-2 cells grow rapidly on the surface of the CNC-PVA hydrogel, confirming that this biomaterial has the potential to be used as a corneal implant [138].

## 4. Toxicological Evaluation and Potential Limitations of NCs-Based Materials

### 4.1. Toxicological Evaluation of NC-Based Materials

Toxicological studies aim to provide data that predict the possible effects on the human health of people exposed to a particular substance, thus reducing the risk of disease.

Limited data on the toxic effects of NCs-based materials (single NCs or NCs in nanocomposites) on human health and important issues such as their biodurability and high aspect ratios make the toxicological assessment of these materials particularly important and at the same time highly necessary. Toxicological testing of NC materials has proved to be particularly difficult, given that there are a wide variety of raw materials, different methods for obtaining each type of nanocellulose, as well as various processes for the preparation of NCs-based materials (hydrogels, films, particles, composites, etc.). All these aspects show the particularly wide range of physicochemical properties of NC-based materials, which must be taken into account when evaluating the toxicological properties. Toxicological data to date show contradictory results related to cytotoxic and genotoxic effects, of NC-based materials, both in vitro and in vivo.

#### 4.1.1. In Vitro Cytotoxicity Analysis

The toxicity of nanocellulose materials is an important factor to be taken into account, especially for the case when the desired application is pharmaceutical or medical. An NCs-based material must be carefully evaluated regarding the impact on human health before marketing it, and for this purpose, the most used assay to evaluate the biocompatibility is in vitro cytotoxicity analysis. The predictive value of this test is usually performed until 92 h and is evaluated by measuring the toxic effect of the NCs-based materials on cells, more exactly the direct or indirect damaging effects on the DNA or chromosomes of cells, a fact that affects the basic functions of cells and determines the death of cell through apoptosis or necrosis. The material’s toxicity also plays a critical role in the precursor materials and the chemicals used in the preparation of the materials.

The toxicological evaluations of NCs-based materials were performed predominantly in vitro, taking into account several parameters, such as the activation of the immune response and inflammation, oxidative stress, and genetic damage of cells. Although in vitro tests do not take into account the complex response of the whole organism and do not allow a direct translation of the results in human health, they still allow an assessment of the interaction between material and cellular components, leading to an imbalance of cell homeostasis and thus, to a measurable effect [179].

As a result of the increased interest of the researchers in order to use NCs materials in drug-delivery applications or in tissue engineering, more exactly of materials prepared only from CNC, CNF, or BNC, or of NCs mixed with different polymers in nanocomposite hydrogels, several publications evaluated the cytotoxicity of them and reported the absence of cytotoxic effects or slight cytotoxic effects.

Thus, for in-depth study of this aspect, the next part briefly summarizes some of the most relevant and recent studies related to the cytotoxicity of each type of NCs, and the results are structured in three tables, presented as follows: the toxicological evaluation of CNC/CNC in nanocomposites in Table 4, in Table 5 of CNF/CNF in nanocomposites and in Table 6 of BNC/BNC in nanocomposites, noting that some of the studies mentioned in the tables have already been presented in Section 3.

The cytotoxicity of cellulose nanocrystals (CNC) prepared from softwood pulp and further derivatized with different carboxyl contents, varying from 6.6 to 1.7 mmol/g, were tested on four different tissue cells line, such as CaCO-2 (human colon epithelial), HeLa (human cervix epithelial), MDCK (canine kidney epithelial) and J774 (mouse ascites macrophage), over a wide range of concentrations (50–300 lg/mL) [180]. The purpose for which the authors chose these types of cancer cells as an assess the relevance of the delivery of nanodrugs for cancer therapy is the fact that: (i) macrophage cells are part of the human body’s immune system, that play a role in eliminating nanocarriers; (ii) nanoparticles accumulate in the kidneys before to be eliminated from the body; (iii) the human colon is part of the GI tract that is used in the administration of drugs; and (iv) HeLa cells are considered a general model for absorption. The authors reported an increase in in vitro cytotoxicity as a result of the influence of each type of cell line, more evident for the MDCK cell line, which exhibited the most marked decrease in metabolic activity (more than 75%) and 50% for the others cell lines. Moreover, a charge-dependent decrease in mitochondrial activity for CNC with a high charge density (>3.8 mmol/g) was observed. It was confirmed the uptake of CNC by cell lines without any damage of membrane or change in cell density.

Catalán et al. [181] demonstrated that CNC obtained from cotton cellulose, with an average length of 135 ± 5 nm and a width of 7.3 ± 0.2 nm, is neither genotoxic nor immunotoxic, under the conditions tested. The cytotoxicity was evaluated in human bronchial epithelial BEAS-2B cells exposed to a concentration range of 15–300 lg/mL for 4, 24, and 48 h and was reported a 55% cytotoxicity at approximately 100 µg/mL. Moreover, CNC did not trigger an inflammatory response in human monocyte-derived macrophages.

Slight cytotoxicity was observed in the case of pristine CNCs when was used in murine alveolar macrophages (MH-S). It was observed that CNC exposure alone does not induce cytotoxicity at low concentrations (1.5 and 5 μg/cm^2^), although a high dose of CNCdry induced a slight decrease in cell viability. It was observed that CNC-based hydrogels did not affect cell proliferation, although, when using the highest hydrogel concentration (90 μg/cm^2^), there was a slight reduction in cell number [182].

The cytotoxicity evaluation of CNC from raw rubberwood and Kenaf-bast fiber showed negligible cytotoxicity at lower concentrations of macrophages (RAW 264.7) and HaCaT cells, up to 700 μg/mL [183]. The cell viability in the medium was mainly dose-dependent. Thus, at concentrations of 300–1000 µg/mL, the viability of RAW 264.7 cells were not affected when exposed to K-CNC, while for the case of R-CNC, the viability of the macrophages was improved. For a concentration of 100 µg/mL, a two-fold increase in cell growth was observed for K-CNC, but at an increased K-CNC concentration, a decrease in viability was noted. The HaCaT cells viability increase by 1.5-fold when was exposed to R-CNC with a concentration of 100 µg/mL.

The cytotoxicity of cellulose nanofibrils (CNF) prepared from Norway spruce (*Picea abies*) with diameters of 10–70 nm and lengths of a few microns, in six different concentrations (31.25 µg/mL–1 mg/mL) was tested on L929 cells, an immortalized fibroblast cell line [184]. It was demonstrated that none of the CNFs induced cytotoxicity and oxidative stress in the L929 cells or necrosis and apoptosis of rat thymocytes or human peripheral blood mononuclear cells (PBMNCs). At a higher concentration (250 µg/mL–1 mg/mL) was recorded a slight inhibition of the metabolic activities of the L929 cells and consequently an inhibition of proliferation. Menas et al. [185] studied the cytotoxicity of two types of CNFs, CNF-gel, and CNF-powder, on the human lung epithelial cells (A549), which were exposed for 24 and 72 h, when was observed a decrease in cell viability more significantly after 72 h (40%–50%), as compared to 24 h (10%–20%). Moreover, both CNFs showed dose-dependent responses (A549 cells were exposed to three different concentrations, 1.5, 15, and 45 μg/cm^2^), with the highest dose causing a decrease in viability of 70% compared to control.

No cytotoxicity was also detected in indirect and direct tests with CNF prepared from Curauá fibers (*Ananas erectifolius L. B. Smith*) in Vero cells by using a cytotoxicity assay according to ISO 10993-5, recommendations (CCIAL 057) [186]. Moreover, suitable biocompatibility was recorded for all the tested protocols (direct and indirect test) due to the mild chemical treatments applied to the fibers, which provided nanoparticles with a nontoxic surface, and during the adhesion test, the cells demonstrated higher affinity to the CNF surface.

Lopes et al. [187] investigated the cytotoxicity of CNFs prepared from a bleached sulfite softwood dissolving pulp with two different surface modifications, carboxymethylated CNF (A-NFC) and hydroxypropyltrimethylammonium CNFs (C-NFC), by using two different assays, Alamar blue (AB) and enzyme lactate dehydrogenase (LDH) assays. It was observed that the introduction of surface-charged groups did not induce cytotoxicity in human lung fibroblast (MRC-5) cells, THP-1 macrophages, and human dermal fibroblasts (HDF). Moreover, no significant differences in total cell number (total LDH) between treated and non-treated cells, nor altered morphology for any of the three cell types treated with NFC gels after 24 h exposure, were found. On the other hand, Lopes et al. [188] studied the NFC materials with different surface modifications and the effect that could induce these modifications on different in vitro biological responses. For this study, five materials were selected, such as unmodified NFC (U-NFC) prepared by enzymatic pretreatment of the wood pulp, A-NFC, C-NFC, phosphorylated NFC (P-NFC), and sulfoethylated NFC (S-NFC). The human epithelial colorectal adenocarcinoma cells Caco-2 response to the samples was evaluated after 24 and 48 h by the cell metabolic activity (using the resazurin assay) and the cell morphology and cell membrane integrity (by using the live/dead staining). It was demonstrated that the metabolic activity of the cells was not significantly affected by the presence of the NFC materials (concentration range 500–50 μg/mL), and no signs of toxicity in terms of cell membrane damage were observed. In vitro toxicology of the same five CNFs-modified samples was also investigated by Aimonen et al. [189] studied the same materials on human bronchial epithelial (BEAS-2B) cells, when observed that all NFCs samples, regardless of the surface functionalization with different surface charges, showed low cytotoxicity, which did not exceed 10%. Moreover, unmodified NFC (U-NFC) and carboxymethylated NFCs (C-NFC) were able to increase intracellular reactive oxygen species (ROS) formation.

In order to develop wound dressings capable of modulating the chronic wound environment, Blasi-Romero et al. [190] selected the thiol-containing amino acid cysteine to endow wood-derived cellulose nanofibrils (CNF) with bioactivity toward the modulation of ROS levels and protease activity. The cytotoxic evaluation was performed in adult human dermal fibroblasts (hDF) by using presto blue (PB) assay, and the results revealed that after 24 h exposure to the CNF materials (concentration range 0.1–1 mg/mL), the cell metabolic activity was above the 70% cytotoxicity limit described by the ISO standard 10993-5. Furthermore, by the exposure to increasing concentrations of carboxylated CNF (c-CNF) or cysteine-functionalized CNF (cys-CNF), the cell morphology was not affected. In addition, cys-CNF demonstrated a dual action in vitro: inhibition of metalloproteinase and radical scavenging activity, which are expected to contribute to the restitution of physiological conditions in the wound bed.

The cytotoxicity of bacterial nanocellulose (BNC) was evaluated by Zikmundova et al. [191] on a bacterial nanocellulose hydrogel loaded with curcumin and its degradation products (DC: vanillin, tumerone, and feruloylmethane), in view of the potential application in wound dressing. In vitro tests performed on human dermal fibroblasts revealed that the cytotoxicity of the materials depended strongly on the concentration of the antibacterial agents and that the degradation products of curcumin are more cytotoxic than pure curcumin. For the same application, such as antibacterial and biocompatible wound dressing, Orlando et al. [192] was used two different active agents, namely glycidyl trimethylammonium chloride (GTMAC) and glycidyl hexadecyl ether (GHDE), for chemical functionalization of bacterial cellulose. Cytotoxicity studies on keratinocyte (HaCaT cell line) cells revealed the nontoxic effect of hydrogels, even after 6 days of contact. There were no changes in the morphology, growth pattern, and cell size, and the in vitro scratch test showed that the samples did not interfere with the healing process.

Pinto et al. [193] were evaluated the cytotoxicity of BC from sugar cane in cultured C3A hepatoma cells (HepG2/C3A), the acute toxicity in adults Wistar rats, the genotoxicity, and antigenotoxic effects. No cytotoxicity or death was recorded for the treatment with the BC in a single oral dose (2000 mg/kg body weight). Moreover, BC attenuated cyclophosphamide (CP)-induced myelotoxicity and genotoxicity; inhibition the incidence of MNPCE (female: 46.94%; male: 22.7%) and increased the ratio of PCE/NCE (female: 46.10%; male: 35.25%).

Khan et al. [194] developed a novel nanocomposite scaffold based on BCN for bone tissue engineering applications. The cytotoxicity of the bacterial cellulose/β-glucan biocomposite scaffold, reinforced with hydroxyapatite nanoparticles (n-HAp) and graphene oxide (GO), tested on mouse pre-osteoblast (MC3T3-E1) cells, revealed that all samples had suitable potential for cell adhesion and proliferation, with very low cytotoxicity, and the minor differences between the samples are attributed to the differences in physicochemical behavior of the scaffolds.

The comparative cytotoxic effects of the NCs materials on human health and the environment are largely unknown, especially hazards and the identification of daily risks from occupational exposure. Nanocellulose materials are often considered materials with no cytotoxicity and immunogenicity or with low cytotoxicity and immunogenicity.

Considering the toxicity evaluation performed on various CNC materials (wood and non-wood), it was observed that most of the studies indicate low or even negligible cytotoxic effects [118,133,138,161,179]. However, there are also some studies in literature that record a cytotoxic effect of CNC [179,180,183,195].

Yanamala et al. [196] showed that particle size and morphology of CNCs may be critical factors affecting the type of immune inflammatory responses. Thus, by exposing to C57BL/6 mice of two processed forms of CNC derived from wood: CNCS (10 wt%; gel/suspension of CNC) and CNCP (freeze-dried powder of CNC), demonstrated that the treatment with CNCS and CNCP induce different cytotoxicity effects, as a result of differences in the dimensions of CNCS and CNCP. Moreover, dose-dependent cytotoxicity and pulmonary damage were observed.

The NCs’ dose-dependent cytotoxic effects have also been investigated by other researchers who confirmed this dependence [182,183,197]. Similar results were obtained by Menas et al. [185] when studied the cytotoxicity potential of five different NCs materials: CNC gel (10% wt.), CNC spray-dried powder (CNC SD), CNC freeze-dried powder (CNC FD), NCF gel (0.9% wt.), and NCF freeze-dried powder (NCF powder)]. CNF samples showed clear dose-dependent and also time-dependent responses in respect to the cytotoxic potential on A549 cells (more significant decrease in cell viability) and in the marker of oxidative stress induction (significant decrease in glutathione (GSH) levels). Even if NCF were more toxic compared to CNC materials, related to cytotoxicity and oxidative stress responses; however, the CNC caused an inflammatory response with significantly elevated inflammatory cytokines/chemokines compared to NCF. Park et al. [198] studied the immunological responses upon 14 days of pulmonary exposure to CNF and CNC, indicated a dose-dependent effect (40 and 80 mg per mouse) on bronchoalveolar lavage (BALF) cytokines and cellular compositions, but with no indications of specific on local and systemic immunotoxicity in the acute response phase. Yanamala et al. [199] demonstrated that the cytotoxic and inflammatory responses upon exposure to different CNMs (CNF and CNC powders materials and lignin-coated materials, L-CNC or L-CNF), isolated from woody biomass, are cell-type specific and dependent on the type, size, and morphology of the each CNMs. Thus, the exposure of the A549 cells of all CNMs, even at the highest concentration (300 μg/mL), more than 90% remain viable for both time points exposure (24 and 72 h). Contrary, a dose-dependent and even a time-dependent was recorded when exposure THP-1 cells to CNMs (viability loss at 72 h, 300 μg/mL, CNC = L-CNC = L-CNF > CNF), indicating that the size, the lignin-coating, as well as the type of CNM are all factors that influence the viability in THP-1 cells.

Several studies evaluated the influence of surface charges of NCs materials on cytotoxicity effects when considered for medical applications [161,180,187,189]. CNC with different amounts of carboxyl groups have demonstrated a charge-dependent decrease in mitochondrial activity for higher charge content [180]. Moreover, it was demonstrated that the introduction of surface charge on NFC gels suppressed the proinflammatory response of THP-1 macrophages when treated with unmodified NFC gels [187]. The surface charge of NCs may affect the interaction of the nanomaterials with proteins and membranes by increasing the risk of inflammation and lung injury. Thus, surface chemistry, surface charge, and functionalization of NCs in order to improve their hydrophobicity can hinder the nanocellulose uptake and the interaction of its functional groups with the cell membrane, affecting their biological responses.

NCs-based hydrogels are suitable for in vivo applications, such as drug and cell delivery, but in close contact with cells, these can become toxic due to mechanical stress of cells and the subsequent death. To evaluate the influence of the mechanical properties of hydrogels prepared from both CNC and CNF on cytotoxicity, two different studies were performed. Meschini et al. [118] show that the mechanical shearing of CNC-based hydrogels on confluent cell monolayers caused a severe reduction in cell viability, regardless of the type of cation used as a crosslinking agent. Moreover, the authors observed that cell viability is dependent on cation concentration, a high concentration causing a decrease in cell survival, toxicity that also depends on cation valence, so divalent cations (Ca^2+^ and Mg^2+^) are more toxic than monovalent ones (Na^+^). In addition, cationic nanoparticles are more cytotoxic than neutral or anionic nanoparticles [200]. Contrary, Basu et al. [27] investigated CNF-based hydrogels on adult human dermal fibroblasts (hDF) cell cultures under the same crosslinking conditions (Ca^2+^) as that of CNC, and it was observed that it induces only small interruptions of a layer of fibroblast cells. The differences between the behaviors of the two types of hydrogels are given by their mechanical properties; namely, CNF-based hydrogel has storage values and elastic modules, which are an order of magnitude larger than those made of CNC, with a comparable concentration of cellulose [201].

Bacterial cellulose is at the forefront of regenerative medicine due to key features such as low toxicity, increases cell adhesion, promotes cell proliferation, migration, and differentiation, allows absorption of blood and exudate, has low tissue adhesion and thermal insulation, which leads to a rapid wound healing [121,202]. BNC is considered to be one of the most suitable materials for wound dressing and skin tissue engineering due to its special properties, such as chemical purity, superior mechanical properties, water-absorbing capacity, nontoxicity, and a lower inflammatory response evaluated by the number of neutrophils, lymphocytes, and macrophages, in studies of BNC-treated mice [107]. At the same time, bacterial cellulose does not show toxic effects on the liver and kidney, as revealed by the levels of alanine transaminase, aspartate transaminase, alkaline phosphatase, blood urea nitrogen, creatine, and lactate dehydrogenase in the blood serum [203].

In conclusion, after an overall toxicological in vitro evaluation of CNC and CNF materials, it has been found that a great influence on their cytotoxicity is given by the particle size and shape, as well as their surface charge and chemistry and their concentration (dose-dependent). In addition, the differences that may occur in the toxicity results of NC-based materials may be due to the type of cells used in the absorption of NC-based materials. Cytotoxicity tests have a high sensitivity due to the fact that the tested cells are isolated in cultures and also lack the protective mechanism that assists the cells in the body. A mammalian cell culture medium is one of the preferred ones because it is a physiological solution capable of extracting a wide range of chemical structures, not just water-soluble ones [204]. However, it has been observed that each cell type has its own function and thus may have a different response when it comes in contact with an NCs-based material, compared to another cell type exposed to the same material. In addition, it was observed that the most commonly used cell line is the BALB/c 3T3 A31 mouse fibroblast cell line, which is a fibroblast cell culture established from disaggregated tissue of an embryonic albino Swiss mouse (Mus musculus) [204].

**Table 4:** Cytotoxicity evaluations in in vitro studies induced by CNC- and CNC-based materials.

Material	Cellulose Source	Toxicological Experiment	Cells Lines	Toxicological Results	Results and Possible Application	Ref.
**CNC**						
CNC	Cotton (Whatman 1 filter paper)	MTS assay; ATP assay.	BEAS 2B hMDMs	Cytotoxicity at 100 mg/mL; No micronuclei induction after exposure to 2.5–100 mg/mL; No induction of proinflammatory cytokines in hMDMs.	Toxicity impact on lungs or bone marrow	[181]
CNC CNC- carboxyl groups	Softwood cellulose pulp	MTS assay	CaCO-2, HeLa, MDCK, J774	CNC not exhibit any significant cytotoxicity; can exert stress on cells if they possess a high charge density; Charge-dependent decrease in mitochondrial activity (charge contents > 3.9 mmol/g).	Drug delivery	[180]
c-CNCs t-CNCs	Cotton Tunicate from *Stuela clava*	LDH assay	A549 MDM MDDC	The aspect ratio in combination with CNCs dose influences the uptake by the 3D co-culture system of the human epithelial airway barrier system.	Toxicity impact on lungs	[205]
CNC	Wood pulp	TB assay	A549	CNC were nontoxic to A549 cells; CNC induced a robust inflammatory response; CNC particles induced a more robust inflammatory response compared to NCF.	Comparable toxicity of CNC with CNF	[185]
CNCgel CNCdry	Wood pulp	LDH assay	MH-S	Low conc. (1.5 and 5 μg/cm^2^) induce no cytotoxicity; A high dose of CNCdry induced a decrease in cell viability; CNC exposure further altered the secretion of cytokines.	Toxicity impact on lungs	[182]
CNC	Wheat bran	MTT assay	Caco-2	Dose-dependent decrease in cell viability, but only with significant results above 1000 μg/mL; The cell viability decreased significantly upon contact with CNC90 (88.09%) at 2000 μg/mL, although CNC30 (92.81%) and CNC60 (93.11%) did not significantly decrease the cell viability.	Biocompatible nanocomposites	[197]
K-CNC R-CNC	Rubberwood fiber Kenaf-bast fiber	MTT assay	RAW 264.7 HaCaT	Cytotoxicity of K-CNC and R-CNC is not significant up to 700 μg/mL; K-CNC and R-CNC induced the formation of ROS in RAW264.7 macrophages.	Biocompatible nanocomposites	[183]
CNC CNC-FL CNC-HM	Cellulose pulp	MTT assay	ATCC PCS201012, A375	No cytotoxicity in direct and indirect contact assays.	Drug delivery	[118]
**CNC in nanocomposites**						
Collagen/CNCs/ GMs scaffolds	MCC	MTT assay	HUVECs	No cytotoxicity; Excellent biocompatibility.	Vascular TE	[133]
CNC CNC-AEM CNC-AEMA	Softwood pulp	ATP assay	J774 A.1 PBMNC	One cationic CNC induced secretion of proinflammatory cytokine IL-1b associated with increase mitochondrial-derived ROS and extracellular ATP levels.	Drug and DNA delivery systems	[206]
PLA/CNCg-PEG nanocomposites	Southern pine	Live/dead assays	hMSCs	Suitable biocompatibility; Nontoxic effect on hMSCs proliferation.	Bone TE	[134]
TEMPO-CNC reinforced PVA hydrogels	MCC	AB assay	HCE-2cells	Nontoxicity; Excellent biocompatibility; The HCE-2 cells viability above the 70%.	Ophthalmic applications	[138]
PVA/CNC nanocomposites	Sugarcane bagasse	MTT assay	L929	Noncytotoxic effect; Strong attachment and proliferation of human fibroblast skin cells on the scaffold.	TE scaffolds	[131]
GA-HA-CNC hydrogels	MCC	CCK-8 assay	NIH-3T3	Cell viability, at 1, 4, and 7 days, higher than 70% limit; No foreign body response.	Skin TE	[132]
CS/Gel/NCC/CP nanocomposites	Soft wood cellulose fibers	MTT assay	Fibroblast cell	Lack of cytotoxicity after 3 days of increasing the cells’ viability.	Wound healing	[113]
CNC/PVA	MCC	AB assay	HCE-2	Nontoxic and cytocompatible profile of the CNC-PVA hydrogel; Suitable biocompatibility toward HCE-2.	Ophthalmic applications	[207]

Abbreviations: BEAS 2B—Human bronchial epithelial cells; hMDMs—Monocyte-derived macrophages; Caco-2—Human colon carcinoma cells; HeLa—Human cervix; MDCK—Dog kidney; J774—Mouse macrophages; A549—Epithelial cells; MDM—Human blood monocyte-derived macrophages; MDDC—Dendritic cells; MH-S—Murine alveolar macrophages; RAW 264.7—Macrophages; ATCC PCS201012—Primary human fibroblasts; A375—Malignant melanoma cells; J774A.1—Mouse monocyte/macrophage; PBMNC—Peripheral blood mononuclear cells; hMSCs—Human mesenchymal stem cells; L929—Mouse fibroblast; NIH-3T3—Fibroblast; HCE-2—Human corneal epithelial cells; LDH assay—Lactate dehydrogenase cytotoxicity assay; MTT assay—(3-[4,5-dimethylthiazol-2-yl]-2,5-diphenyltetrazolium bromide) assay; TB assay—Trypan blue exclusion staining cell viability assay; MTS assay—CellTiter-Glo luminescent cell viability assay; MCC—Microcrystalline cellulose.

**Table 5:** Cytotoxicity evaluations in in vitro studies induced by CNF- and CNF-based materials.

Material	Cellulose Source	Toxicological Experiment	Cells Lines	Toxicological Results	Results and Possible Application	Ref.
**CNF**						
CNF	Bleached dissolving pulp Norway spruce (*Picea bies*)	MTT assay [^3^H]-thymidine uptake assay	L929; Thymocytes PBMNCs	CNFs were not cytotoxic; CNC has non-inflammatory and on-immunogenic properties.	Implantable biomaterials TE	[184]
CNF	*Pinus radiata* pulp	LDH assay MTT assay	HEK NHDF	No toxic effect for keratinocytes and fibroblasts; Non-immunotoxic.	Wound dressings	[208]
CNF CNC	Wood pulp	TB assay	A549	CNF caused a significant decrease in cell viability, at 72 h; Decrease in GSH levels after exposure to CNF.	CNC toxicity	[185]
U-NFC A-NFC C-NFC	Never-dried bleached sulfite softwood dissolving pulp	AB assay LDH assays	HDF MRC-5 THP-1	No cytotoxicity for treated NFC; HDF and MRC-5 cells: the metabolic activity of the treated cells was comparable to that of the negative control; THP-1 cells: a higher metabolic activity of the NFC-treated; U-CNF has an inflammatory response, which was suppressed when surface charges were introduced on the CNFs.	Toxicity impacts on dermal, lung, and macrophage cells	[187]
CNF	Bleached Eucalyptus Globulus kraft pulp	MTT assay	A549 THP-1	Cytotoxic effect at the highest dose tested; Genotoxic effects in A549 cells in the co-cultures; No oxidative DNA damages.	TE	[209]
CNF	Curauá fibers (*Ananas erectifolius L. B. Smith*)	Cytotoxicity assays ISO 10993-5	Vero	CNF shows no cytotoxicity and suitable biocompatibility; The morphology and basic functions of the cells are not affected by the direct contact with the tested materials.	Scaffold TE	[186]
CNF	Softwood bleached kraft fiber	LDH assay	Caco-2, HT-29MTX Raji B	Minimal or no cytotoxicity in a cellular model of the intestinal epithelium (for CNC-25 at 0.75% and 1.5% *w*/*w*, as well as for CNF-50 at 0.75% *w*/*w*).	Biocompatible material	[210]
CNF	Banana peel bran	MTT assay	Caco-2	CNF conc. < 500 mg/mL are not cytotoxic to Caco-2 cells; Viability of Caco-2 decreased with increasing CNF conc.	Biocompatible material	[211]
U-NFC A-NFC C-NFC P-NFC S-NFC	Never-dried bleached sulfite softwood dissolving pulp	Resazurin Assay	Caco-2	None of the NFCs inducing cytotoxic effects in the intestinal cells; The differences in physics-chemical properties of the studied NFCs were not reflected in the Caco-2 response in terms of metabolic activity and cell membrane integrity.	Drug release in gastrointestinal tract (GIT)	[188]
U-NFC C-NFC H-NFC P-NFC S-NFC	Never-dried bleached sulfite softwood dissolving pulp	MTS Assay	BEAS-2B	No cytotoxicity for the highest tested dose (500 μg/mL) for any of the NFCs; None of the NFCs induced genotoxic effects; All samples were able to increase intracellular formation of ROS.	In vitro toxicity of NFCs	[189]
c-CNF cys-CNF	Never-dried bleached sulfite softwood dissolving pulp	PB assay	hDF	cys-CNF did not induce toxic effects on hDF when tested at a concentration up to 0.5 mg/mL, nor did the starting material c-NF cys-CNF presented a dual action in vitro: inhibition of metalloproteinase and radical scavenging activity.	Wound dressing	[190]
**CNF in nanocomposites**						
CNF L-CNF CNC L-CNC	Dissolving pulp	AB assay	A549 THP-1	Cytotoxic and inflammatory responses were dependent on type, size, and hydrophobicity Low or inexistent toxicity of all CNMs in A549 cells Dose-dependent cytotoxic and inflammatory responses in THP-1 cells.	TE	[199]
CNF /GEL/ApA	Bleached birch pulp	MTT assay	MSCs	CNFs and CNF-COOHs have no cytotoxicity; CNF-COOH-ApA cells expressed a low level of stress, visible through lower cell density and the cell inclusions.	Bone TE	[130]
NFC hydrogels crosslinked with Ca^2+^	Bleached sulfite softwood pulp	AB assay	hDF	Cell viability about 78% indicates no toxic effects. No inflammatory response of blood-derived mononuclear cells was observed in relation to the cytokines secretion.	Wound healing	[27]
TEMPO-CNF hydrogel	Bleached birch kraft pulp	MTT assay	hDF	Nontoxicity effect and great hDF cells viability.	Wound healing	[172]
NFC/QCRs nanocomposites	Brown algae	MTT assay	L929	Cells viability is higher than 80% (for 5 to 1000 μg/mL CNFs/QCRs), indicating that there is no cytotoxicity.	Wound healing	[119]

Abbreviations: PBMNC—Human peripheral blood mononuclear cells; HEK—Human epidermal keratinocytes; NHDF—Normal human dermal fibroblasts; A549—Human lung alveolar epithelial cells; HDF—Human dermal fibroblasts; MRC-5—Human lung fibroblast cell line; THP-1—Human monocytic leukemia cell line; A549—Human alveolar epithelial cell line; Caco-2—Human epithelial colorectal adenocarcinoma cells; BEAS-2B—Transformed human bronchial epithelial cells; hDF—Adult human dermal fibroblasts; MSCs—Mesenchymal stem cells; L929—Mouse fibroblast cells; GSH—Levels of glutathione (marker of oxidative stress); GIT—Gastrointestinal tract; MTT—Methylthiazolyldiphenyl-tetrazolium bromide; AB assay—Alamar blue assay; MTS assay—CellTiter-Glo luminescent cell viability assay; PB assay—Presto blue assay; AB assay—Alamar blue cell viability assay; U-NFC—Unmodified NFC; A-NFC—Carboxymethylated-NFC; C-NFC—Hydroxypropyltrimethylammonium-NFC; P-NFC—Phosphorylated NFC; S-NFC—Sulfoethylated NFC; C-NFC—Carboxymethylated-NFC; H-NFC—Hydroxypropyltrimethylammonium-NFC; c-CNF—Carboxylated CNF; cys-CNF—Cysteine-functionalized CNF; L-CNC—Lignin-coated-CNC; L-CNF—Lignin-coated-CNF.

**Table 6:** Cytotoxicity evaluations in in vitro studies induced by BNC- and BNC-based materials.

Material	Cellulose Source	Toxicological Experiment	Cells Lines	Toxicological Results	Results and Possible Application	Ref.
**BNC**						
BNC scaffolds	*G. xylinus*	CCk-8 assay	HUVECs, SMCs, Fibroblasts	BC tubes have no toxic or side effects on vessel-related cells cultured on their surface; the surface of BC tubes was beneficial for cell attachment, proliferation, and ingrowth.	Vascular TE	[161]
Octenidine-loaded BNC	*K. xylinus*	ATP assay	HaCaT	Pure BNC has no influence on HaCaT viability; OCT/BNC extracts exhibited time and concentration-dependent toxicity; cell-damaging effects were observed at extract conc >10% and longer incubation times (24 and 48 h).	Active wound dressing	[101]
BNC	Sugar cane molasses	LDH activity	HepG2/C3A	BC is not cytotoxic (conc. < 170 μg/mL); BNC has a protective effect against CP-induced myelotoxicity and enotoxicity.	Biomaterial TE	[193]
Vaccarin- loaded BNC	*G. xylinus*	MTT assay	L929	BNC-Vac has lower toxicity and better biocompatibility than BNC; RGR for both BNC and BNC-Vac was above 74%.	Wound dressing	[110]
Gentamycin-loaded BNC	*K. xylinus*	NR assay	U2-OS	No cytotoxicity on osteoblast culture after 24 h; gentamycin released from G-BNC after 8 h (400 mg/L) and 16 h (600 mg/L) is enough to eliminate *S. aureus* and *P. aeruginosa* biofilms.	Bone regeneration TE	[212]
Curcumin- loaded BNC	*K. xylinus*	MTS assay	HNDF	The cytotoxic effect on the cells depended on the conc. of curcumin; at 0.5 mg/mL C, a strong cytotoxicity for BNC-C and BNC-DC180; BNC-DC300 suitable cytotoxicity, even at higher extract conc.	Wound dressing	[191]
BNC-GTMAC BNC-GHDE	*G. xylinus*	AB assay	HaCaT	No cytotoxicity; Suitable wound closure rates in the presence of the samples, with complete coverage of the scratched area after 5 days.	Wound dressing	[192]
**BNC in nanocomposites**						
BNC/ALG bilayer composites	*G. xylinus*	ISO10993-5:2009	hNCs hMNC	The composites were found to be noncytotoxic, with a cell viability of 98% and a uniform distribution of cells on the entire porous layer.	Neocartilage TE	[147]
BNC-COL-Ap composites	*G. xylinus*	MTT assay	Osteoblastic cells	The composites did not exhibit cytotoxicity effects.	Bone regeneration TE	[144]
ALG/BCN/COL composite	*A. xylinum*	CCk-8 assay	MC3T3-E1 hAMS	MC3T3-E1 and hams cells were viable and proliferate well, after 2 and 5 days of incubation—suitable cytocompatibility.	TE	[140]
BC-PHEMA composites	*A. xylinum*	AB assay	rMSCs	BC-PHEMA composites are nontoxic and biocompatible; did not influence the morphology and proliferation of the rMSCs.	Wound dressing	[213]
BC/COL composites	*G. xylinus*	Live/ Dead assay	UCBMSCs	No cytotoxicity; Provide advanced microenvironment for UCB-MSCs viability and in vitro proliferation; Significantly elevated proteins and calcium deposition.	Bone regeneration TE	[214]
GEL/BNC nanocomposite	*A. xylinum*	MTT assay	HEK293	BNG showed negligible cytotoxicity.	Wound dressing	[215]
BNC-GEL nanocomposite	-	MTS assay	MRC-5	The samples have no cytotoxicity, and the cells retained their morphology in direct contact with the membrane, The cells attaching to the GEL porous site, while not attaching to the GEL thin-coated BC side.	Bone regeneration TE	[216]
Chitosan-BNC	*K. xylinus*	MTT assay	GM07492	No cytotoxicity for the BC group and BC-Chi-Cip group; Ciprofloxacin-loaded BC-Chi samples exhibited a significant but slight decrease in the metabolic activity of cells (moderate cytotoxicity).	Wound dressing	[217]
GO/n-HAp/BNC/b-glucan biocomposite	-	NR assay	MC3T3-E1	All samples had suitable potential for cell adhesion and proliferation with very low cytotoxicity The order of the cell viability: BgC-1.4 (93%) > BgC-1.3 (79.8%) > BgC-1.2 (71.4%) > BgC-1.1 (68.9%).	Bone regeneration TE	[194]

Abbreviations: HUVECs—Human umbilical vein endothelial cells; SMCs—Smooth muscle cells; HaCaT—Human keratinocytes cells; HepG2/C3A—Human C3A hepatoma cells; L929—Mouse skin fibroblast cells; U2-OS—Osteoblast cell; HNDF—Human neonatal dermal fibroblasts; MC3T3-E1—Mouse osteoblastic cells; rMSCs—Mouse mesenchymal stem cells; UCBMSCs—Human umbilical cord blood-derived mesenchymal stem cells; MRC-5—Normal lung tissues cells; GM07492—Human fibroblast cells; CCk-8 assay—Cholecystokinin-octopeptide proliferation assay; ATP assay—Adenosine triphosphate assay; LDH assay—Lactate dehydrogenase assay; NR assay—Neutral red assay; MTS assay—CellTiter 96^®^ Aqueous Non-Radioactive Cell Proliferation assay; AB assay—Alamar blue assay; *G. xylinus*—*Gluconacetobacter xylinus*; *K. xylinus*—*Komagataeibacter xylinus*; *A. xylinum*—*Acetobacter xylinum*; BNC- GTMAC—BNC functionalized with glycidyl trime-thylammonium chloride (GTMAC); BNC-GHDE—BNC functionalized with glycidyl hexadecyl ether (GHDE).

#### 4.1.2. In Vivo Cytotoxicity Analysis

The in vivo toxicological studies, along with in vitro ones, are necessary to predict the effects of exposure to cellulose nanoparticles on human health, to reduce the risk associated with this unintentional or coincidental exposure, and last but not least, to design safe materials for use in pharmaceutical/medical applications [179]. Using only the cellular models based on viability and oxidative damage responses alone may not be efficient in predicting the toxicity of cellulose nanoparticles [185]. While in vitro systems are useful and suitable for a rapid assessment of nanomaterial effects, in vivo models provide a more thorough understanding of how engineered nanomaterials structures interact with complex biological systems [218].

To date, there are several studies on the toxicity and proinflammatory activity of nanocellulose materials in vivo. A relatively recent literature study of Roman [219] deals extensively with the pulmonary, oral, dermal, and cytotoxicity of cellulose nanocrystals. Although the number of available studies is limited until that date, one of the main findings of the study is that the in vivo toxicity of nanocelluloses (NCs) depends on several parameters that must be taken into account: the exposure method (i.e., ingestion, direct skin contact or inhalation), the exposure duration or the dose of NCs used for animal exposure. Table 7 presents some results of the relatively recent studies on in vivo toxicity tests, using the three different types of nanocellulosic materials, i.e., CNC, CNF, and BC, respectively. The selected results highlight the influence on the immune response of the nanocellulose type (e.g., different morphology, crystallinity, presence of functional groups), the exposure methods (inhalation or ingestion), the time of exposure (24 h, a few days or weeks), the dose administered (single or repeated dose), the mice sex exposed to nanoparticles, etc.

One of the parameters that influence the NCs’ toxicity and that must be taken into account is their morphology, which depends strongly on the cellulose source and preparation procedure [219]. For instance, the influence of morphology on the cytotoxicity and immunogenicity of cellulose nanoparticles was confirmed by the in vivo experiments performed on mice when observed different immune responses to NFC and CNC, respectively [107]: a significantly increased inflammatory response of proinflammatory cytokines and chemokines was obtained by exposure to CNC compared to NFC, although in vitro studies NFC showed a more pronounced response regarding the cytotoxicity and oxidative stress in human lung epithelial cells A549 compared to CNC [185]. However, in the study conducted by Park and coworkers [198], who investigated the effects of four different fibrous materials, the same influence of particle morphology on the immune responses after introduction into the lungs was no longer observed. This time, the NFC turns out to be the nanocellulose that causes an accentuated immune response compared to CNC. In this study, the nanocellulose nanoparticles were administered by oropharyngeal aspiration in BALB/c mice, along with single-walled carbon nanotubes (CNTs) and crocidolite asbestos (ASB), the last two materials being known for their adverse health effects and potential lungs tumor promoters [145,227]. After 14 days of exposure, the expression of cytokines and chemokines in bronchoalveolar lavage (BAL) was quite different depending on the type of nanoparticles to which the mice were exposed: the pulmonary exposure led to discrete local immune cell polarization patterns with a T_H_ 2-like response caused by ASB and T_H_ 1-like immune reaction to NCF, while CNT and CNC caused non-classical or non-uniform responses. Overall, ASB induced a distinct pattern of a much stronger response in terms of immunotoxicity, being the only material not properly handled by the immune system. However, most importantly, nanocellulose fibers (CNF and CNC) did not behave as “asbestos-like” materials, the response to both types of nanocellulose being milder than the response to ASB and CNTs [198].

The results obtained in the study of Shvedova et al. [221] conducted both on male and female mice showed that the mice’s sex influences the response to pulmonary exposure to CNC. The C57BL/6 female and male mice were exposed to 40 μg CNC/mouse by pharyngeal aspiration, two times a week, for three weeks, and the pulmonary outcomes induced by repeated exposure to CNC were investigated. At 3 months post-exposure to CNC, pulmonary inflammation and damage, oxidative stress, elevated TGF-β and collagen levels in lungs were detected, as well as impaired pulmonary functions. All these effects were more pronounced in females compared to male mice. Moreover, sex differences were also detected at the level of global mRNA expression as well as in inflammatory cytokine/chemokine activity.

A remarkable difference in the in vivo nanocellulose fibers toxicity was observed when they are administered in rats by gavage technique. For instance, when Wistar rats were exposed to 1% (*w*/*w*) CNF suspensions two times a week for five weeks, no significant differences in hematology, serum markers, or histology were observed between exposed rats and controls. These results suggest that NC administered orally (ingested) has little acute toxicity and is probably harmless in small quantities [210]. The same aspect is encountered when adult Wistar rats are treated by the same gavage technique, with a single dose of 2000 mg bacterial nanocellulose (BC) per kg body weight per day [193]. In this study, acute toxicity, which evaluates the detrimental effects of a single or several doses administered over a period not exceeding 24 h, was tested using the mouse bone marrow micronucleus (MN) assay. Rats were examined for behavioral changes or any clinical sign of toxicity every 30 min during the first 4 h post-exposure and thereafter once a day for 14 consecutive days. It turned out that the treatment with BNC in a single oral dose induces no signs of toxicity. Moreover, BC caused no alteration of the polychromatic erythrocyte/normochromatic erythrocyte (PCE/NCE) ratio controls and also did not alter the incidence of micronucleated polychromatic erythrocytes (MNPCE) in males and female mice, indicating that this biopolymer was not genotoxic/clastogenic to treated mice [193].

The toxicity of nanocellulose-based materials can be modulated, for example, by modifying the functional groups of NCs (i.e., by carboxylation, carboxymethylation, TEMPO-oxidation, etc.). For instance, Hadrup et al. [223] studied if the pulmonary and systemic toxicity of NFC can be reduced by carboxylation, administered at 6 or 18 μg to mice by intratracheal instillation. Pulmonary exposure to NFC induced pulmonary inflammation and genotoxicity and systemic acute phase response. In addition, non-functionalized NFC administration increased neutrophil influx in BAL and induced acute phase response proteins Serum Amyloid A (SAA3) in plasma. Unlike NFC, carboxylated-NFC produced lower neutrophil influx and systemic SAA3 levels, suggesting that carboxylation of the OH groups reduces the NFC-induced systemic acute phase response. Functionalisation of the OH groups may therefore be considered a strategy to lower the toxicity of these materials [223]. A similar study was performed by the authors of [218], who explore the inflammatory effects of different NFC materials (non-functionalized NFC and functionalized by carboxylation and carboxymethylation, respectively), one bulk-sized cellulose material, and rigid multi-walled carbon nanotubes (rCNT), which is known to cause adverse pulmonary effects similar to asbestos [228]. A total of 24 h after exposure, all tested materials induced a significant influx of neutrophils into BAL. Moreover, rCNT, bulk-cellulose, and non-functionalized NFC, but not carboxymethylated CNF and carboxylated CNF, triggered the recruitment of eosinophils into the airways. Modest immune reactions were also seen after 28 days; however, the effects were markedly attenuated compared to 24 h effects. In contrast to the nanocellulose materials, rCNT induced notable pulmonary inflammation after 28 days. These data indicate that NFC materials do not induce a long-term pulmonary inflammation like rCNT, but still, non-functionalized NFCs are more prone to trigger inflammatory reactions in vivo than NFC functionalized with anionic groups [218].

Another attempt to modulate the toxic effect of NCs was the chemical modification by TEMPO-oxidation of NFC performed by Catalan and coworkers [222]. They examined whether TEMPO-(2,2,6,6-tetramethyl-piperidin-1-oxyl) oxidized NFC, administrated by pharyngeal aspiration, could be genotoxic to mice, both locally in the lungs and systemically in the bone marrow. Female C57Bl/6 mice were treated with different NFC doses, such as 10, 40, 80, and 200 μg/mouse. DNA damage was assessed by the comet assay in bronchoalveolar lavage (BAL) and lung cells, and chromosome damage by the bone marrow erythrocyte micronucleus assay. Significant induction of DNA damage was observed in lung cells, whereas no increase was seen in BAL cells, and no effect was detected in the bone marrow micronucleus assay, either. However, this increase was statistically significant only at the lowest two doses, whereas the higher doses exhibited levels of damage similar to the untreated mice. One explanation for this finding might be that, at the higher concentrations, NFC formed larger agglomerates that could more easily be eliminated from the respiratory tract by coughing. In addition, neutrophilic accumulation in the alveolar lung space was observed with increasing doses. Our findings showed that NFC administered by pharyngeal aspiration caused an acute inflammatory response and DNA damage in the lungs but no systemic genotoxic effect in the bone marrow [222].

### 4.2. In Vivo Degradability of Nanocellulose-Based Materials

Besides biocompatibility and nontoxicity, biodegradability is another requirement for materials with applications in pharmaceutical and biomedical fields [229]. Generally, biodegradability is the property of a material to decompose into simple molecules under the action of biological elements (e.g., enzymes) [230]. Biodegradation is controlled by the material properties (e.g., molecular weight, crystallinity), and thus the biodegradation time can vary from months to years depending on its amorphous/crystalline or hydrophilic/hydrophobic behaviors [231]. For some biomedical applications (e.g., artificial heart valves or menisci), nonbiodegradable materials may be acceptable, whereas, for other applications (e.g., tissue regeneration), the bioresorbable materials that enable tissue regeneration at rates appropriate to their degradation are preferable, biodegradation being an essential property in this regard [232].

NCs are considered to be generally biodegradable because it derives from its natural sources—cellulose. However, there are some distinct differences between cellulose and industrially-obtained NC in terms of their size, morphology, surface characteristics, or physicochemical properties, so it cannot be entirely assumed that nanocellulose has the same biodegradability as cellulose in its native state [233]. Indeed, the studies performed to evaluate the in vivo degradability of nanocellulose showed that its biodegradation still remains a challenge given that NC itself does not undergo complete degradation in the human body due to the lack of specific enzymes [234]. The biodegradation of nanocellulose is usually performed by cellulolytic microorganisms, which produce enzymes (endoglucanases, cellobiohydrolases, and β-glucosidases) that act synergistically, leading to depolymerization of cellulose. In typical enzymatic hydrolysis, endoglucanases and cellobiohydrolases are responsible for the degradation of cellulose to cellobiosis, followed by its hydrolysis to free glucose molecules [235].

Given the facts mentioned above, some efforts have been made to find ways to improve the cellulose fibers’ biodegradation, making them resorbable by the organism [229]. For instance, as demonstrated in an earlier study [236], the incorporation of cellulase enzymes in bacterial cellulose and especially the conjunction of these enzymes with β-glucosidase led to a loss of integrity of almost 80% of tested materials after 2 days. A more recent study showed that the incorporation of the proteinase K, a kind of serine proteases, into scaffolds based on poly(butylene succinate) (PBS)/poly (lactic acid) (PLA) reinforced with NFCs, accelerated the degradation of the NFC-based scaffolds, acting mainly on the C=O bond of PLA. It showed promising prospects in improving the biodegradability of nanocellulose-based scaffolds by incorporating degradable polymers or related enzymes [162].

The introduction of *N*-acetyl-glucosamine residues (GlcNAc) into bacterial cellulose (BC) molecule during its synthesis by metabolically engineered *Gluconacetobacter xylinus* is another attempt that renders the cellulosic biopolymers susceptible to in vivo degradation. It was noticed that the modified-BC with high content of GlcNAc became more susceptible to biodegradation (e.g., to lysozyme and cellulase) compared with BC produced by the control strain. The susceptibility of modified-BC to in vivo degradation was evaluated by its subcutaneous implantation in the female BALB/c mice backs. Visual inspection of implant sites, after 10- and 20-days post-implantation, respectively, indicated little to no degradation of the cellulose produced by the control strain, while the modified-BC was almost entirely degraded after 10 days and completely undetectable after 20 days [237,238].

The oxidation process, using various chemicals, including metaperiodate, hypochlorite, dichromate, nitrogen dioxide, or TEMPO (2,2,6,6-tetramethylpiperidn-1-yl)oxyl) [239,240,241,242,243], makes cellulose materials more vulnerable to hydrolysis and therefore potentially degradable by the human body. The only impediment of this process is that it can significantly alter the structure of cellulose, disturbing the unique, medically desirable properties of cellulose, such as its high tensile strength and conformability. However, this “disadvantage” becomes an “advantage” in soft tissue applications where the material often needs to readily conform to the various contours of the body and to have adequate strength for tissue support and allow easy handling [242].

2,3-dialdehyde celluloses (DAC), obtained via periodate oxidation, have been considered degradable, although only at a slow rate [244]. In tissue engineering applications, cellulose acetate (acetyl cellulose) and ethyl cellulose have hardly been considered as scaffold materials since they are known to degrade very slowly or are practically non-resorbable in vivo. By oxidation with periodate, which has a regioselective action at the C2 and C3 atoms in the glucopyranose units, a structure with two aldehyde groups is formed that is less stable at physiological conditions. Thus, oxidized acetyl cellulose sponges, implanted subcutaneously into Wistar rats in vivo, showed a partial degradation of about 47% after 60 weeks, while for ethyl cellulose the proportion was only 18%) [245]. If the chemical oxidation by is coupled with γ-irradiation, as observed with bacterial cellulose used in soft tissue repair [242], resorbable and fully conformable materials are obtained. In vivo, these oxidized materials showed degradation as early as 2 weeks and continued to be slowly degraded until 26 weeks. A potential mechanism of oxidized-BNC resorption was considered, consisting of two steps: initial rapid resorption via hydrolysis of oxidized domains resulting in degradation of about 70%–80%, followed by the formation of short oligosaccharides that are further cleared by phagocytosis. In this last stage, the action of macrophages and giant cells breaks down the short glucan chains to the point where they can be consumed by the cells [242]. Oxidized bacterial cellulose (OBC) coupled with two degradable polymers, such as chitosan (CS) and collagen (COL), leads to nanocomposites (OBC/COL/CS) with excellent biodegradation. In vivo degradation performance of nanocomposites was evaluated by subcutaneous implantation using male Sprague–Dawley rats and was compared with oxidized regenerated plant cellulose (ORC), which is among the most widely used absorbable topical hemostatic agents for internal bleeding control. ORC has relatively poor biodegradability in vivo. Its complete absorption requires as long as 8 weeks [246]. After implantation for 7 days, sample residues stained purple-brown can be seen on the majority of sections. However, after 30 days, neither residual fragments of samples remaining in the subcutaneous tissue nor an evident pathological response of the implanted sites were observed in nanocomposites groups with oxidized-BC (OBC, OBC/CS, and OBC/COL/CS). In contrast, in the ORC group, even though the fiber diameter decreased markedly compared with that observed after the first 7 days, residual fragments were still found after 30 days [247]. Unlike oxidized-BC, unmodified BC shows poor degradation when is used, for instance, as porous scaffolds for bone tissue engineering, alone or in combination with gelatin via different crosslinking techniques and coating hydroxyapatite. At six weeks after implantation in the nude mice model, the results of the histological analysis showed clearly visible fibers in all scaffolds, suggesting poor degradation of the bacterial cellulose that unfortunately partly inhibited the new bone formation [143].

Similar to bacterial cellulose, the biodegradability of nanocrystalline cellulose (CNCs) and nanofiber cellulose (CNFs), respectively, also evaluated in various studies, is improved by modification (e.g., TEMPO-oxidation) or in combination with other biodegradable polymers. For instance, cellulose nanocrystals combined with biodegradable collagen and gelatin microspheres containing basic fibroblast growth factor (bFGF) were evaluated as a biodegradable platform for long-term release and consequent angiogenic boosting. The scaffolds were implanted into the subcutaneous tissue of the rat backs, and their biodegradability was assessed at 3, 7, and 10 days. With prolonged times, the gross views revealed that scaffolds size was reduced following implantation. On the 3rd day, all of the implanted scaffolds maintained their shape. On the 7th day, the scaffolds based on collagen/CNCs appeared to be smaller in size than other groups. Following that, on the 10th day, no residue of these scaffolds was observed, which may have been caused by degradation in vivo [133]. On the other hand, injectable hydrogels based on TEMPO-oxidized cellulose nanofiber (TOCNFs), methyl cellulose, carboxymethyl cellulose, and polyethylene glycol, with different concentrations (0.2% and 1% *w*/*v*) of TOCNFs were tested regarding their degradation in vivo. The biodegradability was evaluated using male rats by direct dorsal administration of 0.5 mL of injectable hydrogels and observation after two weeks by opening the injection site. Degradation studies revealed that nanocellulose fibers do not undergo complete degradation after 14 days. Moreover, the TOCNFs concentration in the hydrogel was inversely proportional to hydrolytic degradation rate: a higher concentration of TOCNFs in the hydrogel determines a lower degradation capacity, as evidenced by the much smaller size of the hydrogels with 0.2% TOCNFs after 2 weeks [248]. Last but not least, cellulose fibers derived from *Styela clava* tunics (SCT-CF) are also considered slowly degradable compared with wood pulp cellulose fibers (WP-CF). The biodegradability was evaluated by film subcutaneous implantation into Sprague–Dawley (SD) rats for various lengths of time. The weight loss was greater in cellulose films from *Styela clava* (almost 24% of their initial weight) than in films prepared from wood pulp cellulose (less than 10%). It was considered that the higher susceptibility of SCT-CF to degradation would be due to its content of about 98% α-cellulose and very low concentrations of ββ-cellulose and, additionally, a lower crystallinity index of SCT-CF (10.71%) unlike WP-CF (33.78%) [249].

Thereby, it can be concluded that, in terms of biodegradation, the cellulose fibers may be considered nonbiodegradable in vivo or, at best, slowly degradable, but their susceptibility to the action of biological agents can be improved, to a greater or lesser degree, by various methods, i.e., by chemical modification, by association with different well-known degradable polymers or by incorporating of related enzymes. Moreover, biodegradability is a complex characteristic that not only depends on cellulose fiber nature and obtaining methodology but also on its crystallinity form, degree of polymerization, morphology, chemical derivatization, swelling, etc.

## 5. Conclusions

Nanocellulose, one of the most promising green materials of our times, with outstanding properties such as large specific surface area, versatile surface chemistry, and high mechanical properties, has proven to have many applications in various fields, starting from common up to that high-tech biomedical. It is expected that the impressive interest in the development of nanocelluloses (NCs), such as cellulose nanofibrils (CNF), cellulose nanocrystals (CNC), and bacterial nanocellulose (BNC), will continue to grow, and in this respect, it is necessary to update and overcome challenges, such as finding environmentally friendly production processes, with low energy consumption, low costs, and increased production capacity.

Advanced functional materials prepared from NCs alone or by incorporating them into a nanocomposite polymer matrix are the main directions for obtaining adaptable, medically relevant materials. The present review introduces the characteristics of NCs, as well as the main functional materials based on NCs, highlighting the specific properties of each type of nanocellulosic material. It was explored numerous pharmaceutical/biomedical applications of NC-based hydrogels, nanogels, and nanocomposites, starting from the controlled drug delivery to wound dressing and tissue engineering, including scaffolds and medical implants, studies focused on their biocompatibility, biodegradability, and cytotoxicity.

We believe that the systematic analysis of knowledge about nanocelluloses, covering the main cellulose resources, but also recent advances in the last five years in obtaining advanced NCs materials, will open new challenges and potential research directions for pharmaceutical and medical applications.

## Figures and Tables

**Table 1:** Nanocellulose hydrogels/nanocomposites in drug-delivery applications.

NCs Type	Drug Delivery System	Drug	Drug-Release Conditions	Drug Release Mechanism	Ref.
CNC	cCNC/SA double-membrane hydrogels	CH, EGF	PBS, pH 7.4, 37 °C; t_90%_ = 3 days (CH); t_90%_ = 4 to 8 days (EGF).	Swelling/erosion	[89]
	TEMPO-oxidized CNC/CSos	PrHy, IMI	PBS, pH 7.4, room temp.; t_40%_ = 12 min (PrHy); t_80%_ = 2 h (IMI).	-	[90]
	CNC-HDQ complex	HDQ	dH_2_O, room temp., in the dark; t_40%_ = 1 h; t_80%_ = 4 h.	-	[91]
	CS/CNC nanocomposite hydrogels	C	SGF, pH 1.2, 37 °C; 120 min: 65% (0.5% CS/CNC); 50% (2.5% CS/CNC).	Ritger–Peppas model; *n* = 0.61–0.66; Non-Fickian diffusion.	[92]
	QC/cCNC/β-GP nanocomposite hydrogels	DOX	PBS, pH 7.4, 37 °C; t_90%_ = 4 days (0% cCNC); t_90%_ = 7 days (1% cCNC); t_90%_ = 17 days (2.5% cCNC).	Swelling/erosion	[93]
	Gel/CNC nanocomposite hydrogels	TPh	SGF, pH 1.2, 37 °C; 24 h: 90% (5% CNC); 85% (10% CNC); 60% (25% CNC).	-	[94]
	m-CNC/Alg hydrogels	Ibu	PBS, pH 7.4, 37 °C; t = 0–30 min; 45%–60% burst release; t = 30–330 min; sustained release.	Fickian diffusion	[95]
CNF	PDA/TEMPO-CNF composite hydrogels	TCH	PBS; “On-off” drug release under NIR irradiation; 120 min: 60% (pH 5.0); 30% (pH 7.4); 15 h: 70% (pH 5.0); 55% (pH 7.4).	Korsmeyer–Peppas model; Non-Fickian diffusion.	[96]
	CNF/HPMC nanocomposites	KT	PBS, pH 7.4; 8 h: 95% (5% CNF), 62% (0.5% CNF), 56% (0.75% CNF), 37% (1% CNF).	Non-Fickian diffusion; *n* = 0.52–0.61.	[97]
	CNF/Alg hydrogels	MH	SGF, pH 1.2; SIF, pH 7.4, 37 °C; T_40%_ = 90 min (CNF/Alg-50/50, SGF) t_80%_ = 145 min (CNF/Alg-50/50, SIF).	Fickian diffusion mechanism	[98]
BNC	BNC-SA hybrid hydrogels	Ibu	PBS, pH 1.5, 7.0 and 11.8; 37 °C; 24 h: 90% (pH 11.8); 80% (pH 7.0); and 60% (pH 1.5); PBS, pH 7.4; 0.15 V, 0.3 V, 0.5 V; 24 h: 95% (0.5 V); 85% (0.3 V and 0.15 V); 80% (0 V).	Korsmeyer–Peppas model; Non-Fickian diffusion; pH; *n* = 0.498–0.772; E-field; *n* = 0.700–0.491.	[99]
	TCH-loaded BNC composites	TCH	HEPES buffers, pH 7, 37 °C; 3 h: ~100% (free TCH); 90% (0.5% TCH); 60% (0.3% TCH); 20% (0.1% TCH); 10% (0.05% TCH);	-	[100]
	OCT-loaded BNC	OCT	PBS, pH 7.4, 32 °C; 8 h: 82.7% ± 2.6% in first; 24 h 91.8% ± 2.0% after	Ritger–Peppas model; *n* = 0.51–0.55; Non-Fickian diffusion.	[101]
	PI-loaded BNC	PI	PI buffer, 32 °C; t_84%_ = 48 h.	Ritger–Peppas model; *n* = 0.608–0.612	[102]
	PHMB-loaded BNC	PHMB	PHMB buffer, 32 °C; t_87%_ = 48 h.	Ritger–Peppas model; *n* = 0.863–0.871	[102]

Abbreviations: cCNC—Cationic cellulose nanocrystals; SA—Sodium alginate; CH—Ceftazidime hydrate; EGF—Epidermal growth factor human; PBS—Phosphate-buffered saline; CSos—Chitosan oligosaccharide; PrHy—Procaine hydrochloride; IMI—Imipramine hydrochloride; HDQ—Hydroquinone; C—Curcumin; CS—Chitosan; QC—Quaternized cellulose; β-GP—β-glycerophosphate; DOX—Doxorubicin; Gel—Gelatin; TPh—Theophylline; m-CNC—Magnetic cellulose nanocrystals; NIR—Near-infrared spectroscopy; PDA—Polydopamine; HPMC—Hydroxypropylmethyl cellulose; KT—Ketorolac tromethamine; Alg—Alginate; MH—Metformin hydrochloride; SGF—Simulated gastric fluid; SIF—Simulated intestinal fluid; HEPES—(4-(2-hydroxyethyl)-1-piperazine- ethanesulfonic acid); TCH—Tetracycline hydrochloride; AM—Acrylamide; Ibu—Ibupofren; OCT—Octenidine; PI—Povidone-iodine; PHMB—Polihexanide; dH_2_O—Distilled water.

**Table 2:** Drug-loading efficiency of CNC-gelatin hydrogels with different amounts of CNCs. Reproduced with permission from [94]. Elsevier, 2015.

CNC, %	Drug-Loading Efficiency, %
0	64.2
5	65.3
10	55.5
15	51.7
20	44.8
25	41.1

**Table 3:** Nanocellulose hydrogels/nanocomposites in tissue engineering applications.

NCs Type	TE Systems	Applications	References
CNF	CNF/CS nanocomposites	Artificial skin	[30]
	CNF-based thixotropic gels	Breast cancer	[50]
	Double crosslinking 3D-printed CNF hydrogels	Skin TE	[115]
	CNF/PVA bilayer scaffold	Skin TE	[129]
	CNF/Gel/ApA	Bone TE	[130]
CNC	CNC/PVA nanocomposites	Skin TE	[131]
	Gel/HA/CNC hydrogels	Skin wound repair	[132]
	Col/CNC/GMs	Blood vessel	[133]
	PEG-grafted CNC nanocomposites	Bone TE	[134]
	CNC/PVA hybrid hydrogels	Soft TE	[135]
	CNC/PAAm composite hydrogels	TE	[136]
	a-CNC/Gel hydrogels composite	Breast cancer	[137]
	TEMPO-CNC reinforced PVA hydrogels	Corneal implant	[138]
BNC	BNC/Fibrin composites	New blood vessel	[78]
	BNC-Gel/HAp nanocomposites	Bone TE	[139]
	Alg/BNC/Col composite	TE	[140]
	3D BNC/PMS scaffolds	TE; soft tissues regeneration	[141]
	DBC/Col-p	TE; tissues regeneration	[142]
	BNC/PA/Gel/HAp	Bone repair	[143]
	(BNC-Col)-Ap/OGP peptides	Bone TE	[144]
	BNC-CNTs composites	Bone regeneration	[145]
	BNC	Ear cartilage TE	[146]
	BNC/Alg bilayer composite	Neocartilage formation	[147]
	BNC/CS composites	Cartilage tissue regeneration	[148]

Abbreviations: CS—Chitosan; PVA—Poly(vinyl) alcohol; Gel—Gelatin; ApA—Phosphonate; HA—Hyaluronic acid; Col—Collagen; GMs—Gelatin microspheres; PEG—Poly(ethylene glycol); PAAm—Polyacrylamide; a-CNC—Anionic CNC; HAp—Hydroxyapatite; Alg—Alginate; PMS—Paraffin microspheres; DBC—Dialdehyde bacterial cellulose; Col-p—Collagen peptide; PA—Procyanidin; Ap—Carbonate apatite; OGP—Osteogenic growth peptide; CNTs—Carbon nanotubes.

**Table 7:** Cytotoxicity evaluations in in vivo studies induced by NCs- and NCs-based materials.

NCs Type	NC Source	In Vivo Models	In Vivo Experiments	Toxicological Results	Ref.
CNC	Wood pulp	Male C57BL/6 mice, 7–8 weeks old	PA: 40 μg/mouse/day, twice a week, for 3 weeks; Sperm chromatin structure assay and 23-Bio-Plex mouse cytokine assay.	Two-fold increase in HNE–protein adducts levels indicated testicular oxidative damage; Significantly elevated proinflammatory cytokines and chemokines.	[220]
CNC	Wood pulp	Male and female C57BL/6 mice, 7–8 weeks old	PA: 40 μg/mouse/day, twice a week, for 3 weeks; Bio-Plex system: 23-Plex cytokines/chemokines assay and TGF-β 3-plex assay.	After 3 months, pulmonary inflammation and damage elevated TGF-β and collagen levels in lung; Impaired pulmonary functions more pronounced in female compared to male mice.	[221]
CNC, CNF	Wood pulp	Female BALB/c mice, 7–8 weeks old	PA: 40 or 80 μg/mouse; one-time dose; Bio-Plex Pro Mouse Cytokine 23-plex assay.	After 14 days, BALF cytokines and cellular compositions were dose-dependently altered; No specific local or systemic immune cell polarization patterns; No “asbestos-like” behavior of NCs.	[198]
CNF	Softwood bleached kraft fibers	Wistar Han rats, 12 weeks old	G: 1% (*w*/*w*) susp. (10 mL/kg), twice weekly, for five weeks; Pierce LDH assay.	Little acute toxicity and likely “non-hazardous” behavior of ingested NFC in small quantities; Small inflammatory nodules in lungs of rats who had received food with CNF.	[210]
TEMPO- CNF	Spruce sulphite dissolving pulp	Female C57BL/6 mice, 7–8 weeks old	PA: 10, 40, 80 and 200 μg/mouse; one-time dose; Enzyme-linked immune-sorbent assay.	Acute inflammatory response and DNA damage in murine lungs at the lowest doses; The higher doses exhibited levels of damage similar to the untreated mice; No systemic toxicity or genotoxicity in the bone marrow.	[222]
CNF, AS-CNF	Wood pulp	Female C57BL/6, mice, 7–8 weeks old	Intratracheal instillation: 6 or 19 μg/mouse, one-time dose; Comet assay.	Pulmonary inflammation, genotoxicity and systemic acute phase response; Carboxylation reduces systemic acute phase response.	[223]
F-CNF, MC-CNF, AS-CNF, NS-CNF	Wood pulp	Female C57BL/6 mice, 7–8 weeks old	PA: 10, 40, 80 and 200 μg/mouse; one-time dose; Alkaline comet and micronucleus (MN) assays.	DNA damage in lungs, but no systemic genotoxicity for all NFCs, except AS-NFC; F-NFC induces a dose-dependent response in lung cells at 24 h and 28 days post-exposure.	[224]
CNF, MC-CNF, AS-CNF	Wood pulp	Female C57BL/6 mice, 7–8 weeks old	OPA: 10 or 40 μg/mouse; one-time dose; Commercial human IL-1β and TNF-α ELISA kits.	Induced immunity response, after 24 h, for all NFCs; Non-functionalized NFCs caused Th2-type inflammation; Modest immune reactions after 28 days.	[218]
BC	*G. xylinus*	Male C57/Bl6 mice, 9 weeks old	Intraperitoneally injection: 0.5 and 5 mg BC/200 μL PBS. Biochemical parameter tests.	After 7 days, none of the parameters tested (albumin, total cholesterol, aspartate aminotransferase, alanine transaminase and triglyceride) were affected by the BC presence; BC is nontoxic and did not produce any adverse effects in body or organ weight.	[225]
BC	Sugar cane molasses	Adult Webster male and female mice, 85 days old	G: 2000 mg BC 0.8%/kg bw/d, for 3 consecutive days; MN assay.	BC cause no alteration of the PCE/NCE ratio; Protective effect against CP-induced myelotoxicity and enotoxicity.	[193]
BC-, MBC-PP meshes	*A. xylinum*	Han Wistar rats and Albino rabbits, both sexes, 2–3 months old; Dunkin-Hartley guinea pigs, 3 months old	PP meshes, BC-, and MBC-coated meshes implantation into the pocket of panniculus camosus muscles along the dorsal midline for a one-month period.	BC- and MBC-coated meshes evoke the lowest immune response to surrounding tissue after implantation; The tested material neither cause any allergic or significant intradermal reactions nor pathological changes in internal organs.	[226]

Abreviations: PA—Pharyngeal aspiration; G—Gavage; OPA—Oropharyngeal aspiration; HNE—Hydroxynonenal moiety; TGF-β—Transforming growth factor beta; BALF—Bronchial alveolar lavage fluid; LDH—Lactate dehydrogenase; F-CNF—Fine cellulose nanofibrills; MC-CNF—Carboxymetylated-CNF; AS-CNF—Carboxylated CNF; MN—Mouse bone marrow micronucleus assay; PCE—Polychromatic erythrocyte; NCE—Normochromatic erythrocyte; CP—Cyclophosphamide; MBC—Bacterial cellulose modified with chitosan; PP—Polypropylene meshes.

## Data Availability

Not applicable.
